# Integrated Sensing and Communication in a Fiber‐Wireless System With Time‐Phase Synthesis Dimension Waveform

**DOI:** 10.1002/nap2.70290

**Published:** 2026-09-09

**Authors:** Shengkun Chen, Zhenglin Tang, Weiqiang Lyu, Mengke Wang, Yaowen Zhang, Zhen Zeng, Lingjie Zhang, Heping Li, Zhiyao Zhang, Yong Liu

**Affiliations:** ^1^ School of Optoelectronic Science and Engineering University of Electronic Science and Technology of China Chengdu China; ^2^ State Key Laboratory of Electronic Thin Films and Integrated Devices University of Electronic Science and Technology of China Chengdu China; ^3^ Southwest Institute of Technical Physics Chengdu China

**Keywords:** integrated sensing and communication, microwave photonics, stimulated Brillouin scattering, time‐phase synthesis dimension

## Abstract

The convergence of integrated sensing and communication (ISAC) across optical and wireless domains is a cornerstone for next‐generation networks, yet it is hindered by the fundamental incompatibility between fiber and wireless systems. Here, we introduce a unified fiber‐wireless ISAC system driven by a single microwave photonic platform. By leveraging a tailored time‐phase synthesis dimension waveform, our architecture simultaneously delivers high‐speed data transmission, precise wireless ranging, and accurate fiber diagnostics. The system achieves an equivalent common public radio interface (CPRI) data rate of 47.92 Gbit/s, a wireless ranging resolution of 0.29 m, and a fiber delay measurement precision of 56.6 ps capable of breakpoint localization. Furthermore, the synthesized waveform suppresses stimulated Brillouin scattering by 11 dB in high‐power analog fronthaul, paving the way for compact and energy‐efficient base stations. This work provides scalable and power‐efficient framework that bridges the long‐standing gap between optical and wireless realms, setting the stage for future green and intelligent mobile networks.

## Graphical abstract

A unified fiber‐wireless integrated sensing and communication system is enabled by a single microwave photonic platform with a time‐phase synthesis dimension waveform. This architecture simultaneously delivers high‐speed data transmission, high‐precision wireless ranging, and accurate fiber diagnostics over a high‐power analog radio‐over‐fiber link. Raising the Brillouin threshold by 11 dB supports a minimalist base station architecture, paving the way for next‐generation green and intelligent mobile networks.

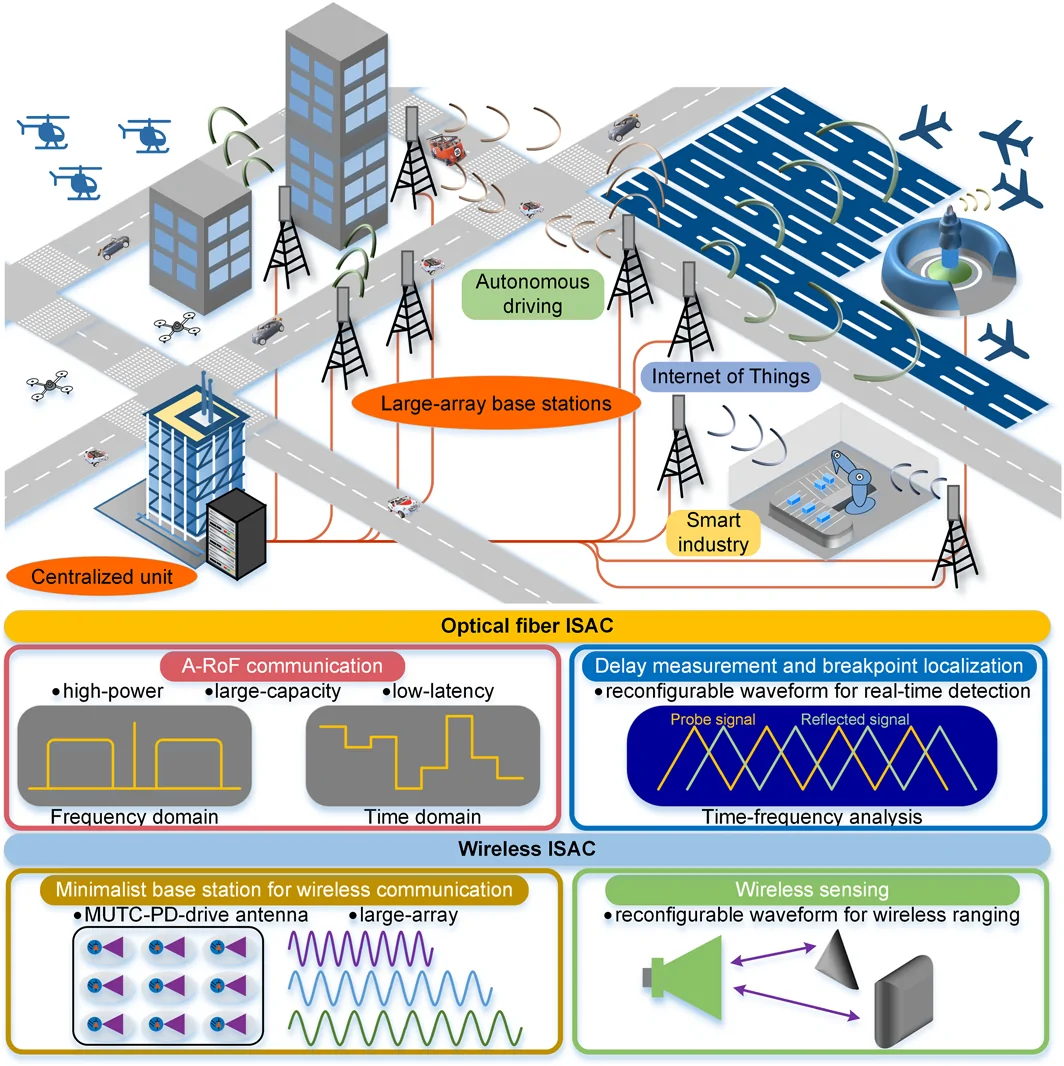

## 1 Introduction

Integrated sensing and communication (ISAC) is emerging as a key enabling technology for next‐generation optical and wireless networks, leveraging shared hardware to consolidate both functions on a single platform [1, 2, 3, 4, 5, 6, 7]. This integration significantly reduces system redundancy and is widely recognized as an energy‐efficient solution for future mobile infrastructure [4, 6]. A critical segment in realizing this vision is the fronthaul, which serves as the last mile of fiber‐wireless convergence and largely dictates overall network performance [8, 9, 10, 11]. Fronthaul supports bandwidth‐intensive and latency‐sensitive applications—such as autonomous driving, smart industry, and the Internet of Things—that require both high‐speed connectivity and real‐time environmental awareness [12, 13, 14].

As illustrated in Figure 1, this vision can be realized through dense deployment of distributed, large‐scale antenna arrays, enabling seamless wide‐area coverage [15, 16]. In such architectures, optical fibers not only transport high‐capacity data to base stations but also provide precise, sub‐nanosecond synchronization to radio‐frequency (RF) front‐ends [17, 18, 19]. Meanwhile, large antenna arrays support joint communication and radar‐like terminal sensing [20, 21]. Together, these elements outline a scalable pathway toward intelligent, energy‐efficient sixth‐generation (6G) mobile networks and beyond [22, 23, 24].

**FIGURE 1:**
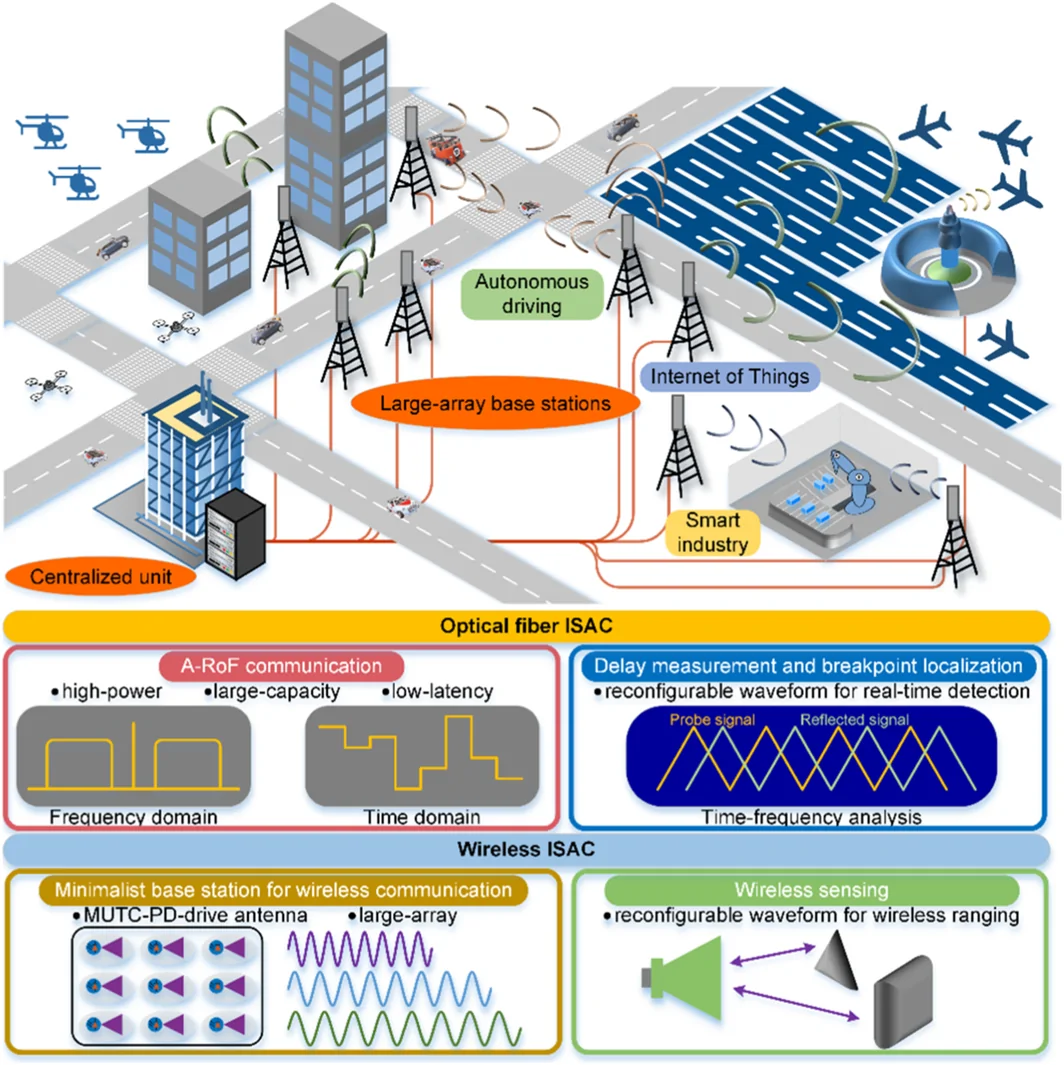
Mobile networks capable of integrated sensing and communication (ISAC) across optical and wireless domains. The system employs a unified time‐phase synthesis dimension waveform over a high‐power analog radio‐over‐fiber (A‐RoF) link to simultaneously enable broadband wireless communication, fiber delay measurement, and wireless sensing, supporting a minimalist base station (BS) architecture. MUTC‐PD, modified uni‐traveling‐carrier photodiode.

The realization of this fiber‐wireless ISAC vision demands a unified and reconfigurable hardware platform capable of performing both optical and wireless functions. However, several critical bottlenecks impede progress [1, 2, 4, 5, 7, 25, 26, 27]. First, broadband communication and high‐precision sensing inherently compete for scarce spectrum resources across both optical and wireless domains. Conventional electronic or photonic solutions only partially alleviate this scarcity within isolated dimensions, lacking a holistic approach [1, 2, 4, 5, 7]. Second, nonlinear effects in optical fibers—particularly stimulated Brillouin scattering (SBS)—severely constrain the transmit power in fronthaul links [25, 26, 27]. This limitation necessitates power‐hungry RF amplifiers at base stations, resulting in an unsustainable architecture for densely deployed next‐generation networks due to excessive energy consumption and bandwidth constraints [28, 29, 30, 31]. These challenges, rooted in the fundamental incompatibility between optical fiber and wireless systems, underscore the limitations of conventional approaches. In this context, microwave photonics provides a promising pathway to bridge this gap, as evidenced by recent demonstrations of on‐chip modules enabling full‐spectrum wireless communications and millimeter‐wave (mmW) radar [8, 32]. Therefore, a holistic microwave photonic solution that simultaneously overcomes the spectral and power bottlenecks is essential to fully harness the potential of ISAC in converged networks.

Here, we demonstrate a microwave photonic‐empowered fiber‐wireless system that seamlessly consolidates communication and sensing across optical and wireless domains. By employing a fast frequency‐swept optical carrier generated from a novel time‐phase synthesis dimension laser, our system simultaneously mitigates SBS—increasing the Brillouin threshold by 11 dB over 11.8‐km‐long fiber link—and enables multifunctional operation. This approach achieves high‐power (> 24 dBm) transmission of optically‐carried wireless communication signals at an equivalent common public radio interface (CPRI) data rate of 47.92 Gbit/s, supporting a minimalist base station with a photodiode‐driven antenna architecture. Concurrently, it delivers precise sensing capabilities, with 56.6‐ps precision in fiber delay measurement and 0.29‐m resolution in wireless ranging. The reconfigurable laser further enables adaptive sensing and fiber breakpoint localization within this unified hardware platform, paving the way for next‐generation green and intelligent mobile fronthaul.

## 2 Results

### 2.1 Principle

Figure 2a illustrates the schematic diagram of our seamless fiber‐wireless ISAC system, which comprises three subsystems: (I) fast frequency‐swept laser, (II) optical fiber ISAC, and (III) wireless ISAC.

**FIGURE 2:**
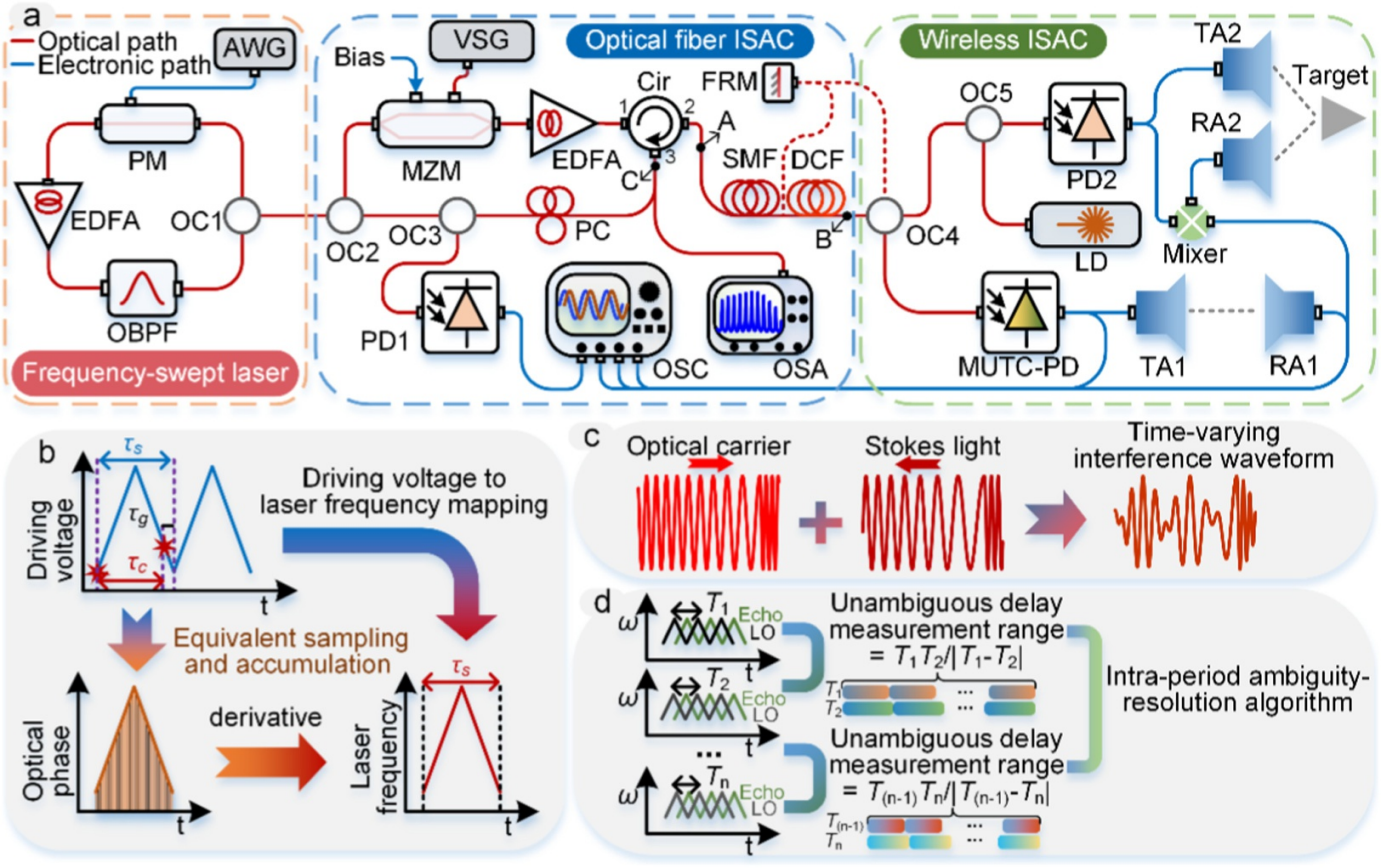
Schematic diagram of the fiber‐wireless ISAC platform. (a) System architecture. An LFM‐CW laser is generated by a time‐phase synthesis dimension laser (see panel b for mechanism) and split for dual use: as an optical carrier for A‐RoF transmission and as a probe for fiber sensing. The laser chirp rate is engineered to suppress SBS and resolve measurement ambiguities (see panels c, d). At the base station, the signal is converted for wireless ISAC. A, B, and C denote the monitor point for measuring injected, output, and back‐scattered optical power, respectively. AWG, arbitrary waveform generator; Cir, optical circulator; DCF, dispersion‐compensating fiber; EDFA, erbium‐doped fiber amplifier; FRM, Faraday rotator mirror; LD, laser diode; MUTC‐PD, modified uni‐traveling carrier photodiode; MZM, Mach–Zehnder modulator; OBPF, optical bandpass filter; OC, optical coupler; OSA, optical spectrum analyzer; OSC, oscilloscope; PC, polarization controller; PD, hotodiode; PM, phase modulator; RA, receiving antenna; SMF, single‐mode fiber; TA, receiving antenna.

In Subsystem I, a linearly frequency‐modulated continuous‐wave (LFM‐CW) optical carrier is generated by a time‐phase synthesis dimension laser. As depicted in Figure 2b, the laser oscillation initiates from the spontaneous emission of an erbium‐doped fiber amplifier (EDFA). The core mechanism employs an electro‐optic phase modulator (PM), which imprints a low‐frequency triangular driving signal from an arbitrary waveform generator (AWG) onto the circulating light within the ring cavity. This process linearly maps the driving voltage to the instantaneous optical frequency, generating the LFM‐CW output. An optical bandpass filter (OBPF) defines the oscillation bandwidth, whereas a polarization‐maintaining architecture ensures operational stability. The bandwidth and chirp rate of the linearly frequency‐modulated optical carrier can be reconfigured by adjusting the repetition frequency and peak‐to‐peak amplitude of the triangular driving signal (see Supporting Information S1 for derivation).

In Subsystem II, the linearly frequency‐modulated laser serves a dual role: as the optical carrier for an analog radio‐over‐fiber (A‐RoF) link and as the probe signal for fiber sensing. Its time‐phase synthesis characteristic underpins two key advancements. First, as shown in Figure 2c, the rapid carrier frequency variation prevents stable interference with back‐scattered Stokes light, thereby suppressing SBS and enabling high‐power transmission. This SBS suppression, in turn, mitigates the dual impairment—broadband noise introduction and optical carrier power depletion—that would otherwise severely degrade the signal‐to‐noise ratio (SNR) of the wireless communication signal (Supporting Information S1) [25, 26, 27]. Second, Figure 2d illustrates a Vernier‐effect‐based sensing scheme, which employs a series of linearly frequency‐modulated lasers with reconfigured periods to achieve large‐unambiguous‐range delay measurement, with intraperiod ambiguity resolved by a dedicated algorithm (Supporting Information S1) [33].

Within the optical fiber ISAC link, the linearly frequency‐modulated laser is split into a local oscillator (LO) for sensing and an optical carrier for communication. The carrier is modulated with a 64‐QAM wireless signal (centered at 6 GHz with a bandwidth of 135 MHz, equivalent to a common public radio interface data rate of 47.92 Gbit/s) using a Mach–Zehnder modulator (MZM). This signal is transmitted over an 11.8‐km fiber link (10‐km standard single‐mode fiber (SMF) + 1.8‐km dispersion‐compensating fiber (DCF)). A portion of the signal is reflected via a Faraday rotator mirror, emulating a fiber breakpoint. The reflected signal is combined with the local oscillator via an optical coupler (OC3), and is detected by a photodetector (PD1). The resulting de‐chirped RF signal is analyzed to determine fiber delay, enabling fiber breakpoint localization through iterative measurement (See Supporting Information S1 for principles).

In Subsystem III, the system extends its functionality to wireless communication and radar‐like sensing. Two optical signals from OC4 are routed for distinct purposes. The first is directly fed into a minimalist base station, where a modified uni‐traveling carrier photodiode (MUTC‐PD) converts the optically‐carried signal into an RF signal for wireless communication via an integrated antenna. The second branch generates a wireless sensing probe through optical heterodyne between the LFM‐CW signal and a CW laser in PD2 [34, 35]. The resulting RF signal, which preserves the linear frequency modulation characteristic, is split into two paths: one is radiated into free space by a transmitting antenna (TA2), and the other serves as a local oscillator reference. The echo signal captured by a receiving antenna (RA2) is then mixed with this local oscillator, and the de‐chirped intermediate frequency (IF) output is acquired for high‐resolution wireless ranging analysis (see Supporting Information S1 for details).

### 2.2 LFM‐CW Laser Generation

The reconfigurable linearly frequency‐modulated laser is characterized via coherent optical detection. As shown in Figure 3a, the laser is mixed with a narrow‐linewidth reference laser using a 90° optical hybrid and a balanced photodetector, converting the optical chirp into a linearly frequency‐modulated microwave signal. The signal is captured by a high‐speed real‐time oscilloscope (256 GSa/s sampling rate, 59 GHz bandwidth), and its time‐frequency distribution—identical to that of the original laser due to the linear frequency mapping—is derived from the Wigner–Ville distribution. Driven by a 4.936 MHz triangular waveform (2.3 Vpp), the time‐phase synthesis dimension laser (free spectral range (FSR): 5 MHz) theoretically yields a laser frequency tuning range of 158 MHz and a chirp rate of 1560 THz/s. Experimentally, the generated microwave signal exhibits a 150 MHz bandwidth (Figure 3b) and a matching 4.936 MHz repetition rate (Figure 3c). The time–frequency distribution in Figure 3f confirms a linear chirp with a 150 MHz span and a rate of 1481 THz/s, slightly lower than theory due to RF loss between the PM and the AWG, yet closely following the scaled driving waveform (purple dashed line). This demonstrates precise waveform mapping and flexible control via a low‐cost approach. Temporal waveforms at key frequencies are shown in Figure 3d, with a full period of 202.6 ns in Figure 3e. High mutual coherence is evidenced by a uniform cross‐correlation trace over 260 µs (Figure 3g–i). The system also allows chirp reconfiguration by adjusting the drive signal for adaptable fiber sensing (Supporting Information S1).

**FIGURE 3:**
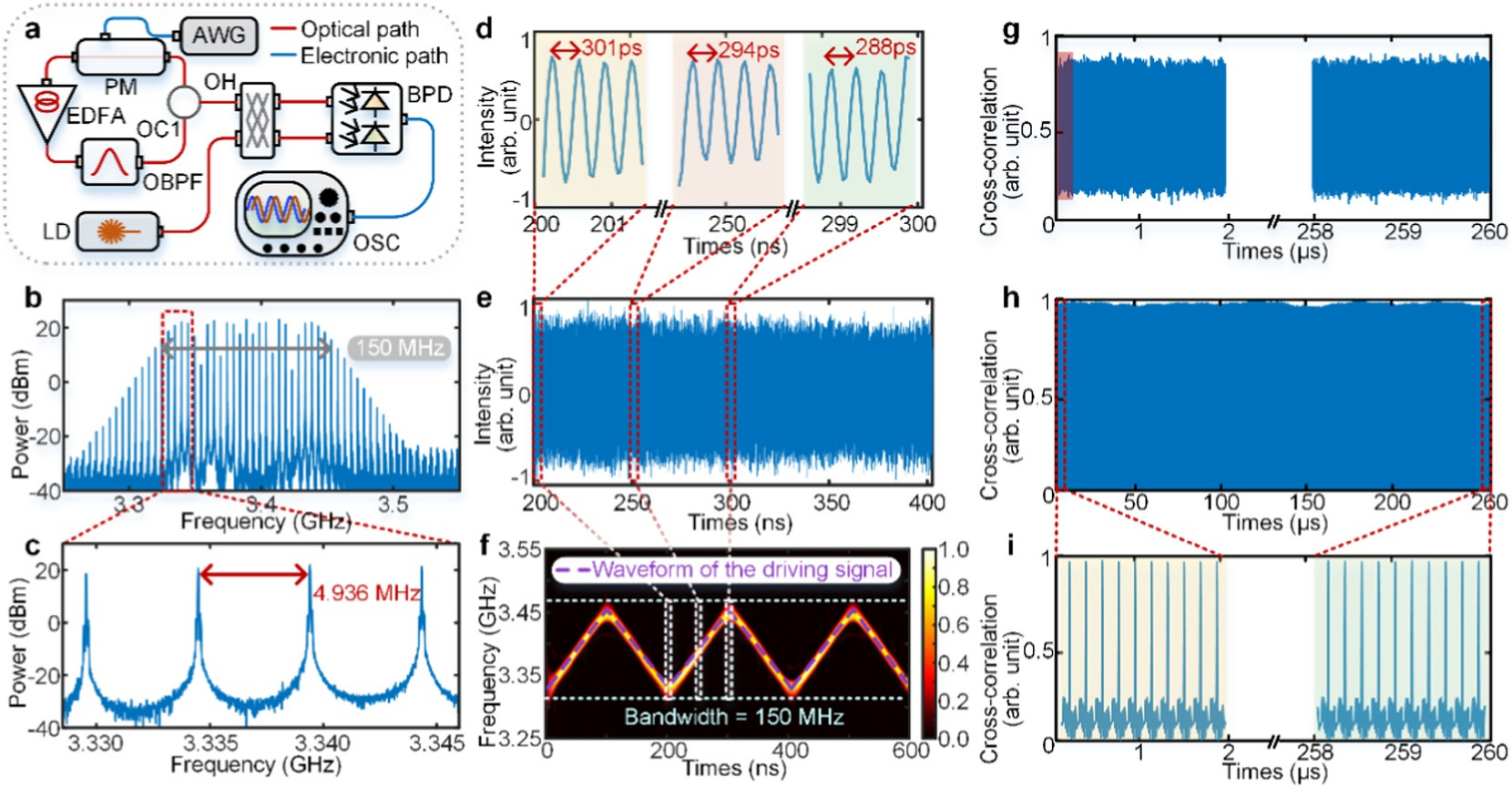
Characterization of the LFM‐CW laser. (a) Heterodyne detection setup for laser characterization. (b) Microwave spectrum. (c) Spectrum detail. (d) Waveform segments. (e) Temporal waveform within ∼200 ns. (f) Time‐frequency distribution. (g) Waveform with 1283 periods for cross‐correlation calculation. The reference waveform is within the red rectangular. (h) Cross‐correlation trace. (i) Cross‐correlation detail at start and end. BPD, balanced photodetector; OH, optical hybrid.

### 2.3 Optical Fiber ISAC

#### 2.3.1 Long‐Haul High‐Power A‐RoF Link With SBS Suppression Supporting Minimalist Base Station

We evaluate the Brillouin threshold of the high‐power A‐RoF link by monitoring optical power at point A (injected), B (output), and C (back‐scattered) in Figure 2a. The linearly frequency‐modulated optical carrier raises this threshold from 12 dBm (conventional CW carrier) to 23 dBm, defined as the input power where backscattered power reaches 1% of input [36, 37]. This 11‐dB enhancement enables linear power growth until the threshold is exceeded (Figure 4a). The suppression reaches 41 dB at 19 dBm input (Figure 4c), effectively curbing SBS until it resurges at 24 dBm (Figure 4d). Although a wider frequency sweep could further raise the threshold, it would compromise signal coherence and period duration, thus limiting broadband and sensing performance—highlighting the need for parameter balancing (Supporting Information S1). Error vector magnitude (EVM) measurements confirm the A‐RoF link maintains < 5% EVM up to 24 dBm (Figure 4e), outperforming the conventional link which degrades above its SBS threshold. Constellations in Figure 4f–i demonstrate robust 47.92 Gbit/s communication without SBS‐induced distortion at high power.

**FIGURE 4:**
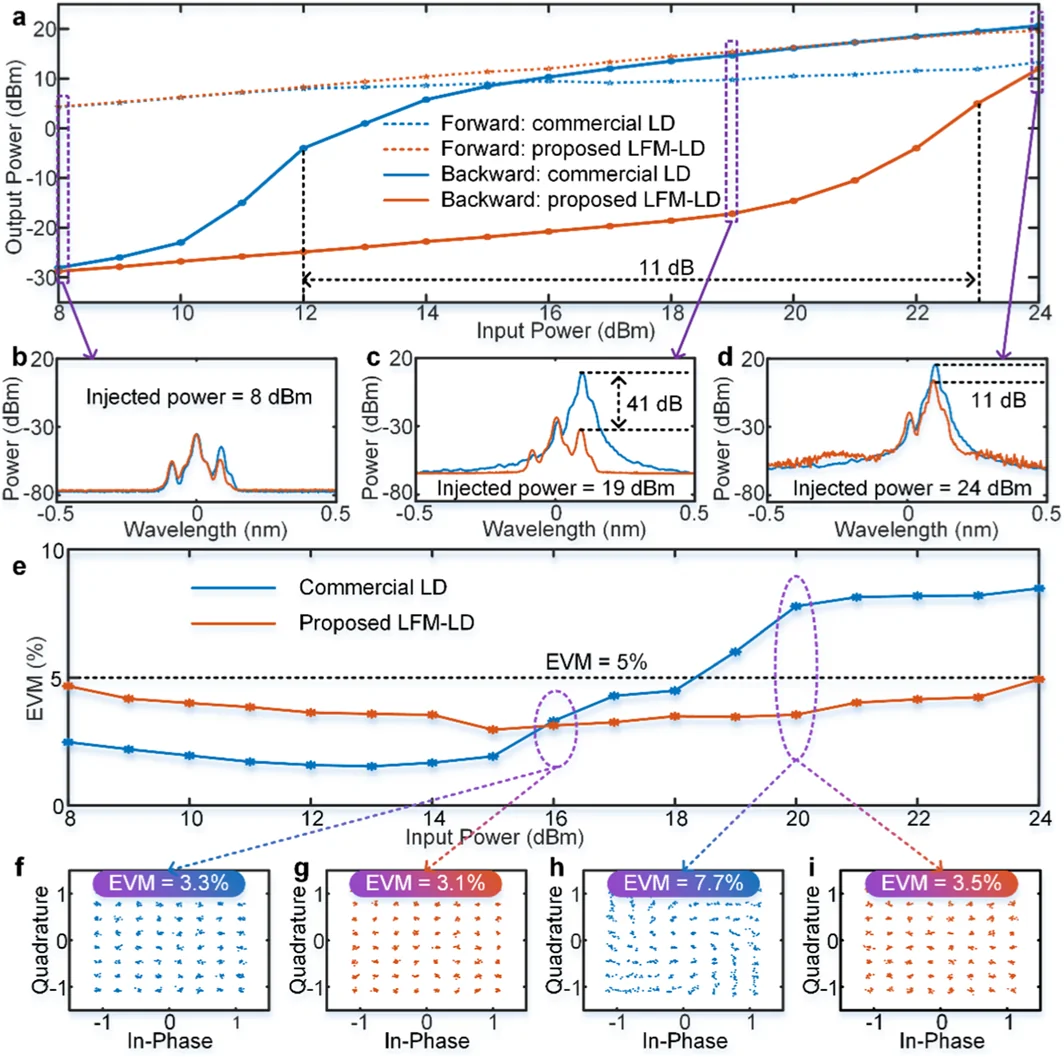
SBS‐suppressed A‐RoF link performance. (a) Forward‐transmitted and backward‐scattered optical powers versus injected power. (b, c, d) Backward‐scattered optical spectra at injected powers of 8, 19, and 24 dBm. (e) EVMs for different injected powers. (f–i) Constellation diagrams of demodulated signals at injected powers of 16 dBm (f, g) and 20 dBm (h, i), comparing conventional (blue) and proposed (orange) schemes.

#### 2.3.2 High‐Precision Time Delay Measurement and Fiber Breakpoint Localization

Figure 5 shows fiber delay measurement results using a reconfigurable linearly frequency‐modulated probe with three periods (*T*_1_ = 202.881 ns, *T*_2_ = 202.593 ns, *T*_3_ = 202.265 ns). Leveraging the Vernier effect, this configuration extends the unambiguous range to 124.931 μs (=min{T1T2/(T1‐T2), T2T3/(T2‐T3)})—sufficient to cover the 11.8‐km fiber link. The time–frequency distributions of the probes (Figure 5a,d,g) confirm the set periods and tailored frequency ranges (118.58–149.51 MHz). De‐chirping yields IFs of 89.812 MHz, 103.553 MHz, and 90.441 MHz (Figure 5b,e,h,c,f,i), from which a final delay of 118.5679 μs is derived. This delay corresponds to a fiber length of 11.8579 km, closely matching the actual length of 11.8 km, with minor discrepancy attributable to component delays without calibration. Allan deviation analysis (Figure 5i) confirms a precision of 56.6 ps at 2.77 μs averaging, validating the suitability of this method for applications such as fiber breakpoint localization (Supporting Information S1).

**FIGURE 5:**
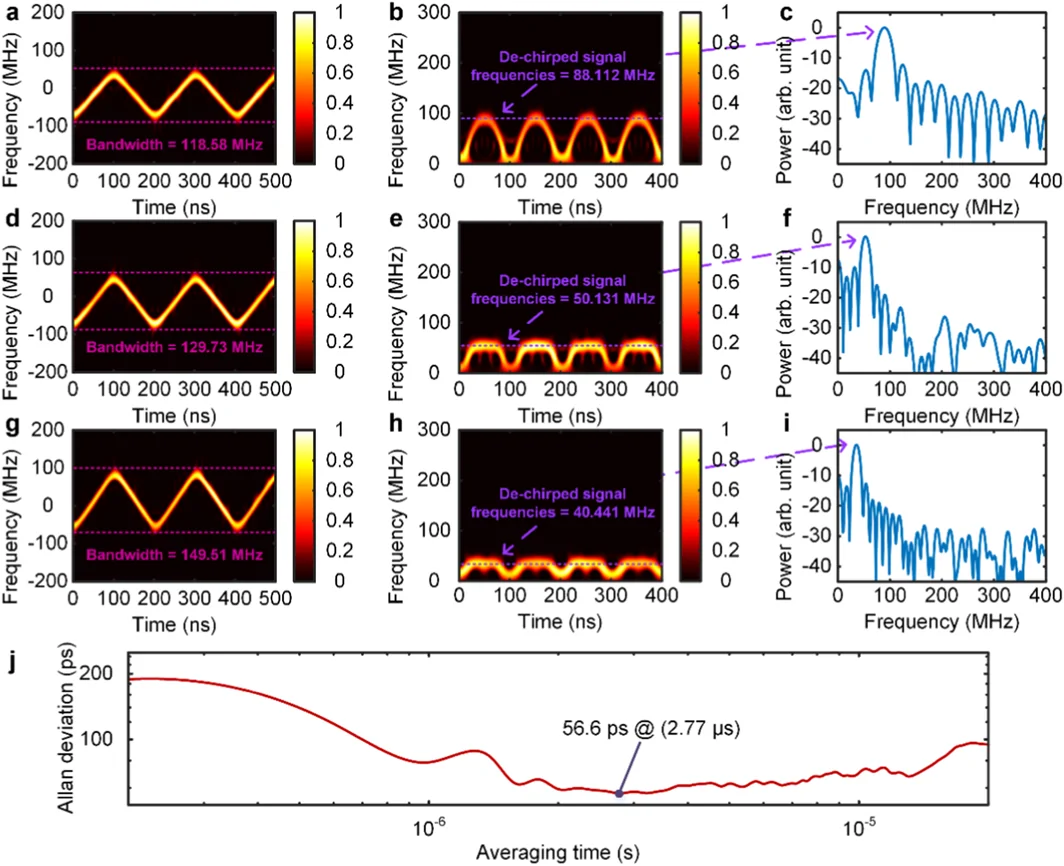
Fiber‐delay measurement results. (a, d, g) Time–frequency distribution of the three linearly frequency‐modulated probe lasers. (b, e, h) Corresponding time‐frequency distributions of the de‐chirped signals. (c, f, i) Measured frequencies of the de‐chirped signals. (j) Allan deviation of the fiber‐delay measurement.

### 2.4 Wireless ISAC

#### 2.4.1 Broadband Wireless Communication via a Minimalist Base Station

We demonstrate broadband wireless communication over the high‐power A‐RoF link using a remote MUTC‐PD‐driven antenna. At a fixed 19 dBm optical power, the EVMs for signals of varying bandwidths—measured under back‐to‐back, fiber‐only, and wireless (3 m) conditions—all conform to the 3GPP 8% threshold (Figure 6a). The corresponding constellation diagrams for the 135 MHz signal remain well‐defined in all scenarios (Figure 6b–d), with minor scattering due to transmission loss, confirming robust link performance.

**FIGURE 6:**
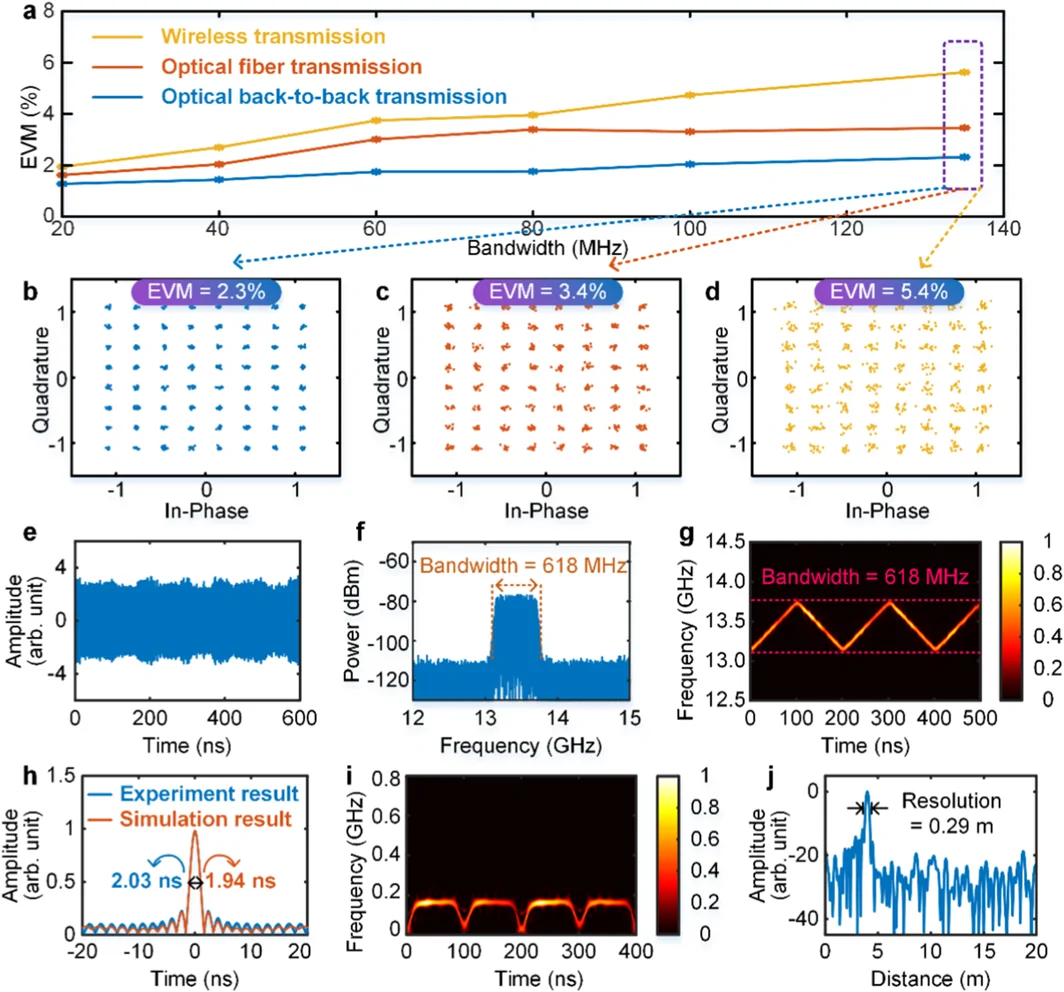
Wireless ISAC results. (a) EVMs of communication signals across different bandwidths and transmission scenarios. (b–d) Corresponding constellation diagrams for a 135‐MHz‐bandwidth signal under back‐to‐back, fiber‐only, and wireless conditions. (e) Temporal waveform of the wireless sensing probe. (f) Spectrum of the generated microwave signal. (g) Time–frequency distribution of the waveform in (e. h) Autocorrelation of the experimental (blue) and ideal (orange) probe signals. (i) Time–frequency distribution of the de‐chirped signal. (j) Wireless ranging result.

#### 2.4.2 High‐Precision Wireless Ranging

The wireless sensing probe generated at the base station (Figure 6e) is characterized as a linearly frequency‐modulated microwave signal centered at 13.5 GHz with a 618 MHz bandwidth and a 200.6 ns period, as evidenced by its power spectrum and time–frequency distribution (Figure 6f,g). Its high quality for sensing is confirmed by an autocorrelation result showing a pulse compression width of 2.03 ns, closely matching the idea value of 1.94 ns (Figure 6h). The de‐chirped signal exhibits stable frequency characteristics (Figure 6i), enabling wireless ranging through frequency‐to‐range mapping (Figure 6j) [38]. The system achieves a ranging resolution of 0.29 m, which can be further optimized by adjusting the bandwidth and period of the linearly frequency‐modulated laser.

## 3 Discussion

In summary, we have demonstrated a fully integrated fiber‐wireless ISAC system centered around a reconfigurable linearly frequency‐modulated laser, simultaneously achieving 47.92 Gbit/s data transmission, 56.6 ps fiber delay measurement precision, and 0.29 m wireless ranging resolution. To our knowledge, this is the first unified fiber‐wireless ISAC system driven by a single microwave photonic platform, and its performance remains competitive even against state‐of‐art representative standalone fiber‐only or wireless‐only ISAC systems (Table 1). The long‐haul, SBS‐suppressed analog fronthaul link further enables a minimalist base station architecture, pointing toward energy‐efficient and scalable mobile networks.

**TABLE 1:** Comparison of representative works in optical fiber and wireless ISAC.

Ref.	Feature	Optical fiber ISAC		Wireless ISAC	
		Communication data rate	Delay measurement precision	Communication data rate	Ranging resolution
This work	A unified fiber‐wireless ISAC system	47.92 Gbit/s	56.6 ps	47.92 Gbit/s	0.29 m
[5]	Optical fiber ISAC only	56 Gbit/s	20 ns	N/A	N/A
[39]		25 Mbit/s	641 ps	N/A	N/A
[40]	Wireless ISAC only	N/A	N/A	4.8 Gbit/s	0.026 m
[21]		N/A	N/A	6 Gbit/s	0.035 m

### 3.1 Comparison of Swept Laser Technologies

The fast frequency‐swept laser source is a key enabler of the proposed system, offering distinct advantages over state‐of‐the‐art alternatives in terms of chirp linearity, sweep speed, reconfigurability, and system complexity, as summarized in Table 2.

**TABLE 2:** Comparison of representative fast frequency‐swept laser generation techniques.

Method	Sweep bandwidth	Sweep speed/Repetition rate	Linearity	System complexity	Ref.
Directly modulated DFB laser	∼GHz	∼MHz (repetition rate)	Nonlinear	Low	[41]
MEMS‐tunable VCSEL	> 50 nm	3.849 × 10^4^ THz/s (chirp rate)	Nonlinear	Medium	[42, 43]
FDML laser	> 100 nm	MHz range (repetition rate)	Good	High	[44, 45, 46]
Time‐stretched swept laser	> 100 nm	8.47 × 10^8^ nm/s (sweep speed)	Good	High	[47, 48, 49]
**This work**	**> 30 GHz**	**> 1.5 × 10^9^ THz/s (chirp rate)**	**Truly linear**	**Low‐medium**	**—**

*Note:* The bold values highlight the exceptional performance of our time‐phase synthesis laser.

These techniques fall into four main categories: directly modulated distributed feedback (DFB) lasers, micro‐electromechanical systems (MEMS)‐tunable vertical‐cavity surface‐emitting lasers (VCSELs), Fourier domain mode‐locked (FDML) lasers, and time‐stretched swept lasers—each with distinct trade‐offs. Directly modulated DFB lasers offer the simplest configuration but suffer from intrinsically nonlinear chirp and limited modulation bandwidth due to the coupled carrier and thermal dynamics [41]. MEMS‐tunable VCSELs achieve wide tuning ranges exceeding 50 nm, yet their electrostatic actuation introduces inherent nonlinearity and restricts the sweep speed to the MHz regime [42, 43]. FDML lasers provide broad bandwidths and MHz‐level repetition rates, but demand precise synchronization, sophisticated dispersion management, and complex feedback control [44, 45, 46]. Time‐stretched swept lasers can reach sweep rates up to 100 MHz with bandwidths exceeding 100 nm. However, this approach requires femtosecond seed lasers and precise dispersion modules, resulting in high system complexity and limited reconfigurability [47, 48, 49].

In contrast, the time‐phase synthesis approach employed in this work simultaneously delivers continuous, truly linear chirp, exceptionally high chirp rates (> 1.5 × 10^9^ THz/s), full reconfigurability of both bandwidth and repetition rate, and low‐to‐moderate system complexity [50, 51]. This unique combination of attributes, not concurrently available in any of the aforementioned alternatives, makes our approach particularly well‐suited for the integrated fiber‐wireless ISAC applications demonstrated here, where high‐speed data transmission, high‐precision sensing, and adaptability across both optical and wireless domains are simultaneously required.

### 3.2 Reconfigurable Characteristic and Integration Potential of the LFM‐CW Laser

The linearly frequency‐modulated laser offers full reconfigurability in repetition frequency, chirp rate, and tuning range, enabling performance tailoring for specific ISAC tasks. This flexibility, however, entails trade‐offs among three key metrics: ranging resolution, SBS suppression, and measurement update rate. For instance, increasing the modulation index widens frequency tuning range and improves resolution, but may introduce nonlinear chirp that compromises ranging precision. Reducing the frequency offset enhances SBS suppression, yet at the cost of an increased risk of mode‐hopping. Raising the repetition frequency of the driving waveform increases chirp rate, benefiting both SBS suppression and sensing speed, but pushes the limits of AWG bandwidth.

The exceptionally high chirp rates achievable (e.g., exceeding 1.5 × 10^9^ THz/s in our setup) further underscore the potential for precision velocity sensing, as the Doppler resolution scales with the chirp rate [50, 51]. Looking forward, migrating the laser source from discrete fiber‐optic components to a photonic integrated circuit (PIC) offers the most promising pathway for further performance enhancement. An integrated cavity with superior mechanical and thermal stability would enable stable operation, thereby significantly increasing the time‐bandwidth product (TBWP). Moreover, optimized modulator designs with reduced optical losses and lower half‐wave voltage could accommodate higher modulation indices without introducing excessive nonlinearity. Such an integrated platform would simultaneously improve ranging resolution, SBS suppression, and update rate, unlocking advanced ISAC capabilities including centimeter‐level resolution, longer‐reach high‐power fronthaul, and real‐time dynamic perception.

### 3.3 Prospects for Integration of the Wireless Base Station

In our current setup, wireless communication and sensing signals are generated at the remote base station using two PDs due to the limited operational bandwidth (∼8 GHz) of the customized MUTC‐PD, which is insufficient for the 13.5 GHz sensing waveform. However, recent advances demonstrate that high‐power MUTC‐PDs can achieve bandwidths up to 100 GHz [52, 53, 54, 55]. Adopting such devices would enable a simplified base station using a single MUTC‐PD‐driven antenna, thereby reducing system complexity and cost.

Besides, the wireless communication distance is constrained by the relatively low RF output power of the MUTC‐PD in the experiment. Under an injected optical power of 19 dBm, the output power of the wireless communication signal is −1 dBm. The directional gain of the commercial antennas in the experiment is 21 dB. The free‐space transmission loss at 6 GHz over 3 m is calculated by using the Friis equation as [36, 56].

(1) $$\text{Loss}=20\times \mathrm{lg}\left(\frac{4\times \pi \times 3\times 6\times 10^{9}}{3\times 10^{8}}\right)=58\;\text{dB}$$

Hence, the received wireless communication signal power from the RF port of the RA is calculated to be −17 dBm (=−1+21−58 + 21), which is almost the lowest power level that can be demodulated in the experiment (Supporting Information S1). Through using the MUTC‐PD with an output RF power level of 1.86 W [21], the wireless transmission range is expected to be extended to over 300 m, fully aligning with the requirements for 6G minimalist base stations [57, 58].

## 4 Materials and Methods

The fiber‐wireless system comprises three main modules: the fast frequency‐swept laser subsystem, the optical fiber ISAC subsystem, and the wireless ISAC subsystem.

### 4.1 Fast Frequency‐Swept Laser Subsystem

The ring‐cavity laser is constructed with a polarization‐maintaining architecture, incorporating an electro‐optic PM (EOSPACE, 2.8 V half‐wave voltage), an erbium‐doped fiber amplifier (EDFA, Amonics) with tunable pump power, a tunable OBPF (Alnair Labs), and a 50:50 OC. A low‐speed arbitrary waveform generator (RIGOL) supplies the triangular driving signal to the PM. Self‐oscillation is initiated and sustained by adjusting the EDFA pump to achieve a net cavity gain larger than 1. The bandwidth of the OBPF is set according to the desired laser sweep range for each experiment. The output linearly frequency‐modulated laser is extracted via OC1 for subsequent use.

### 4.2 Optical Fiber ISAC Subsystem

The linearly frequency‐modulated laser is split via OC2 into two paths, where the lower branch serves as the LO for sensing, and the upper branch functions as the optical carrier. The carrier is modulated by a 64 QAM wireless signal (6 GHz center frequency, 20–135 MHz bandwidth) from a vector signal generator (Rohde & Schwarz, SMW200A) via a Mach‐Zehnder modulator (FUJITSU, FTM7938EZ), amplified by an EDFA (Amonics, AEDFA‐BO‐23), and transmitted over a 10‐km SMF with a group‐velocity dispersion (GVD) coefficient of ∼−21.7 ps^2^/km followed by a 1.8‐km DCF with a GVD coefficient of ∼120.6 ps^2^/km for dispersion compensation. The signal is then split via OC3 into three branches, where the middle and lower branches feed the base station for wireless ISAC, with the lower branch detected by an MUTC‐PD, and the upper branch is reflected by a Faraday rotator mirror. The reflected signal is combined with the local oscillator and detected in PD1 for sensing. All outputs are recorded by a real‐time oscilloscope (Keysight, UXR0594A), with an optical spectrum analyzer (OSA, Yokogawa, AQ6370D) monitoring backscattering. For breakpoint localization, the mirror is repositioned after the SMF to emulate a fiber breakpoint.

### 4.3 Wireless ISAC Subsystem

For wireless communication, a pair of double‐ridged horn antennas (4.6–7.1 GHz bandwidth, 21 dBi gain @ 6 GHz) is used for transmitting and receiving, spaced 3 m apart. The transmitting antenna is directly driven by the MUTC‐PD without any additional RF amplification. For wireless sensing, the probe signal is generated via optical heterodyne (Figure 2) between the transmitted optical carrier and a narrow‐linewidth laser (< 100 Hz linewidth, NKT, BASIK X15), producing a linearly frequency‐modulated microwave signal centered at 13.5 GHz in a customized 20 GHz photodetector. The sensing antennas (11.9–18 GHz bandwidth, 20 dBi gain @ 13.5 GHz) are similarly configured without RF amplifiers.

## Author Contributions

**Shengkun Chen:** conceptualization, investigation, funding acquisition, writing – original draft, methodology, validation, visualization, writing – review and editing, formal analysis, data curation, software. **Zhenglin Tang:** investigation, methodology, validation, writing – original draft, writing – review and editing, software, formal analysis, data curation. **Weiqiang Lyu:** conceptualization, methodology, formal analysis. **Mengke Wang:** conceptualization, validation, formal analysis, project administration. **Yaowen Zhang:** conceptualization, formal analysis, funding acquisition, validation, project administration. **Zhen Zeng:** validation, formal analysis, visualization. **Lingjie Zhang:** validation, formal analysis, visualization. **Heping Li:** conceptualization, validation, formal analysis. **Zhiyao Zhang:** conceptualization, writing – review and editing, validation, project administration, formal analysis, supervision, resources. **Yong Liu:** validation, formal analysis, writing – review and editing, project administration, resources, supervision.

## Conflicts of Interest

The authors declare no conflicts of interest.

## Supporting information


Supporting Information S1

## Data Availability

The data are available from the corresponding authors upon reasonable request.
