# Supplementary material for: Integrated Sensing and Communication in a Fiber‐Wireless System With Time‐Phase Synthesis Dimension Waveform
Source: Nanophotonics. 2026 Sep 9;15(17):e70290. doi: 10.1002/nap2.70290 (PMC13672126; doi:10.1002/nap2.70290)
Supplement: Supplementary file 1 [file NAP2-15-e70290-s001.pdf]

Supplementary Information for
Integrated sensing and communication in a fiber-wireless system with time-phase synthesis dimension waveform

Shengkun Chen[1,2,#], Zhenglin Tang[1,2,#], Weiqiang Lyu[1,2,3], Mengke Wang[1,2,*], Yaowen Zhang[1,2,*], Zhen Zeng[1,2], Lingjie Zhang[1,2], Heping Li[1,2], Zhiyao Zhang[1,2], and Yong Liu[1,2]

[1]School of Optoelectronic Science and Engineering, University of Electronic Science and Technology of China, Chengdu 610054, China

[2]State Key Laboratory of Electronic Thin Films and Integrated Devices, University of Electronic Science and Technology of China, Chengdu 610054, China

[3]Southwest Institute of Technical Physics, Chengdu 610041, China

[#]These authors contributed equally to this work: Shengkun Chen, Zhenglin Tang

[*]Corresponding author: Mengke Wang (wmk5627@uestc.edu.cn), Yaowen Zhang (zhangyaowen@uestc.edu.cn)

Contents

Supplementary note I - Principle of LFM-CW laser generation 2

Supplementary note II - SBS suppression based on LFM-CW laser 5

Supplementary note III - Operation principle of fiber delay measurement and breakpoint localization 10

Supplementary note IV - Operation principle of wireless sensing 15

Supplementary note V - Reconfigurability of LFM-CW laser 18

Supplementary note VI - Theoretical analysis of the coherence-sweep bandwidth trade-off in the time-phase synthesis laser 20

Supplementary note VII - Fiber breakpoint localization 24

Supplementary note VIII - Receiver sensitivity analysis of the wireless link 25

Supplementary note I: Principle of LFM-CW laser generation

Figure S1 shows the schematic diagram of linearly frequency-modulated laser generation. The core lies in the mapping from the driving waveform voltage to the laser frequency based on time-phase dimension synthesis, i.e., equivalent optical phase sampling and accumulation. When the ring-cavity laser operates in free-running state, i.e., without phase modulation, the output is a single-frequency CW laser. Once a driving waveform is applied to the PM, it is sampled by the circulating optical field and accumulated in optical phase with a sampling interval equal to the round-trip time in the ring-cavity laser, as shown in Fig. S1a. Through setting the period of the driving waveform to be slightly offset from the round-trip time with a small difference of τ_g, the driving waveform is sampled by the circulating laser with an equivalent interval time of τ_g. When τ_g is sufficiently small, the proposed scheme physically integrates the driving waveform voltage onto the laser phase, as shown in Fig. S1b. Since laser

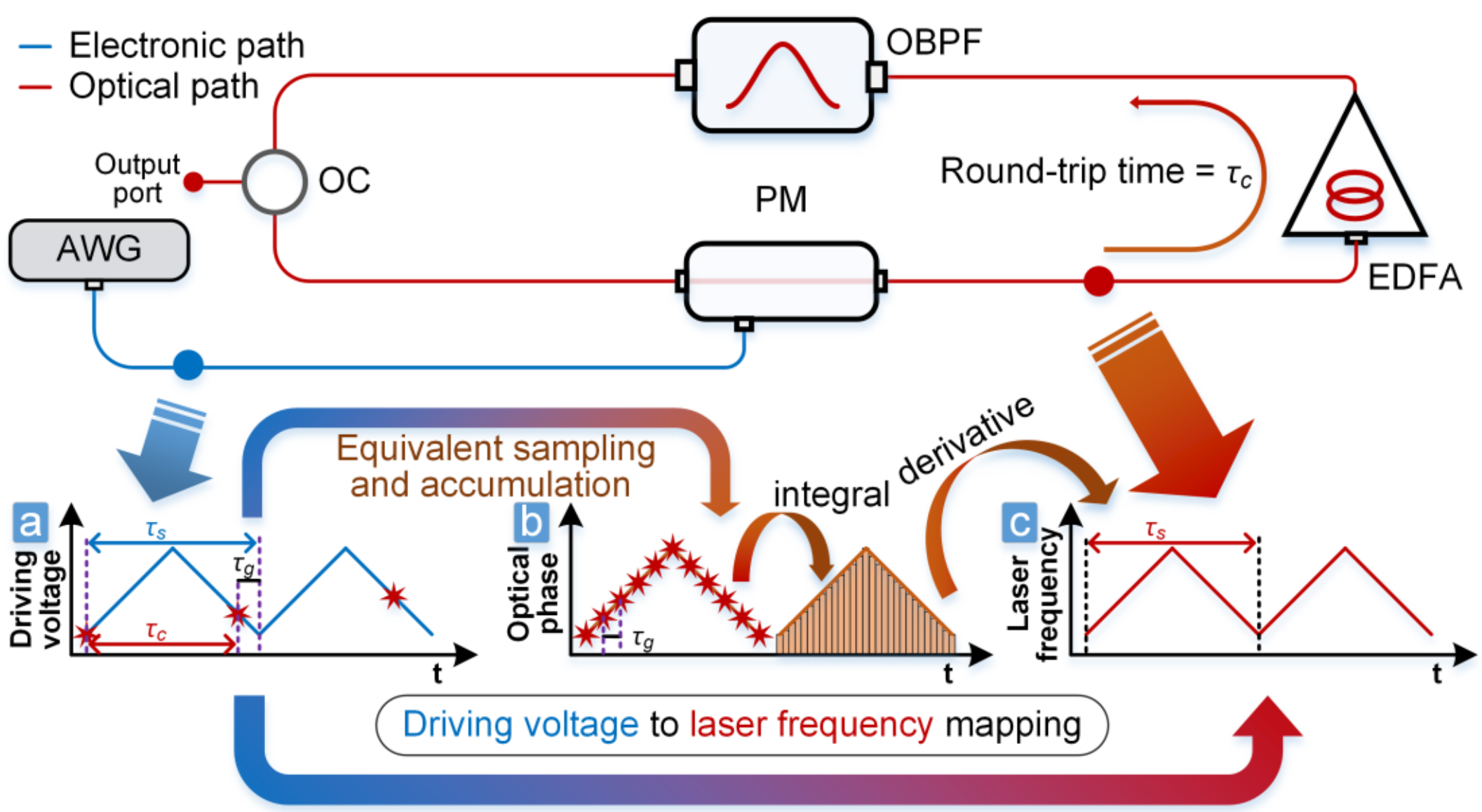

Fig. S1. Schematic diagram of the ring-cavity laser based on time-phase dimension synthesis. The PM in the laser cavity with a round-trip time of τ_c is used as a phase sampler to impose an additional optical phase induced by the Pockels effect on the recirculating optical field. Inset **a** illustrates a triangular waveform with a period of τ_s applied to the PM. Insert **b** presents the equivalent optical phase sampling and accumulation process in the ring-cavity laser. Insert **c** shows the instantaneous frequency of the generated LFM-CW laser, which is linearly proportional to the driving waveform voltage.

frequency is the time derivative of laser phase, the integral-derivative duality inherently maps the driving waveform voltage onto the laser frequency, as shown in Fig. S1c. As a result, linearly frequency-modulated lasers with configurable chirp rates can be generated by tuning the voltage and period of the driving waveform.

Mathematically, the optical field at t_0 can be expressed as $E_0(t_0)\exp(j\varphi(t_0))$, where $E_0(t_0)$ is the input optical field at t_0 before the PM, and $\varphi(t_0)= \pi s(t_0)/V_\pi$ is the PM-induced additional optical phase at t_0. $s(t_0)$ and V_π are the driving voltage of the PM at t_0 and the half-wave voltage of the PM, respectively. After a single round trip in the cavity with a period of τ_c, the laser is phase-modulated by the driving waveform again with a new optical filed of $E_0(t_0)\exp(j\varphi(t_0)+j\varphi(t_0+\tau_c))$. After N round-trips, the output optical field of the ring-cavity laser at t_0 can be expressed as

$$E_{out0}(t_0)=\gamma^N E_0(t_0)\exp\left(j\sum_{n=0}^{N-1}\varphi(t_0-n\tau_c)\right), \quad (1)$$

where γ is the net gain per round trip that is set to be slightly larger than 1 for activating self-oscillation and maintaining continuous sampling. Through setting the period of the driving waveform to be slightly offset from the round-trip time with a small difference of τ_g, i.e., $\tau_g = \tau_c - k\tau_s << \tau_s$, equivalent optical phase sampling with a sampling time interval of τ_g is achieved, where k is a positive integer, and τ_s is the period of the driving waveform. As a result, the output optical field at t_0 can be rewritten as

$$E_{out}(t_0)=\gamma^N E_0(t_0)\exp\left(j\sum_{n=0}^{N-1}\varphi(t_0-n\tau_g)\right), \quad (2)$$

Since $\tau_g << \tau_s$, the PM-induced optical phase accumulation $\Phi(t_0)$ can be expressed as the integral of the additional optical phase to the equivalent sampling time interval, i.e.,

$$\Phi(t_0)=\sum_{n=0}^{N-1}\varphi(t_0-n\tau_g)=\frac{\pi}{V_\pi\tau_g}\left(\int_0^{t_0}s(t)dt-\int_0^{t_0-N\tau_g}s(t)dt\right) \quad (3)$$

Since the PM starts to be injected at $t_0 - N\tau_c$, and $s(t) = 0$ in the interval of $t < t_0 - N\tau_c$, Equation (3) can be rewritten as

$$\Phi(t_0)=\frac{\pi}{V_\pi\tau_g}\left(\int_0^{t_0}s(t)dt-\int_{t_0-N\tau_c}^{t_0-N\tau_g}s(t)dt\right)=\frac{\pi}{V_\pi\tau_g}\int_0^{t_0}s(t)dt-\frac{\pi Nk\tau_s s_0}{V_\pi\tau_g}, \quad (4)$$

where s_0 is the mean value of $s(t)$ over a single period from $t_0 - N\tau_c$ to $t_0 - N\tau_g$. The instantaneous frequency excursion of the output laser can be obtained from the

derivative of the phase to the time, which is calculated as

$$f\left(t_0\right) = -\frac{1}{2\pi}\frac{d\Phi\left(t_0\right)}{dt} = -\frac{s\left(t_0\right)}{2V_\pi \tau_g} \tag{5}$$

Equation (5) can be rewritten as

$$f\left(t_0\right) = -\frac{f_c f_s}{2V_\pi \Delta f} s\left(t_0\right) \tag{6}$$

where $f_c = 1/\tau_c$ is the free spectral range (FSR) of the ring-cavity laser. $f_s = 1/\tau_s$ is the repetition frequency of the driving waveform. $\Delta f = f_s - kf_c$ is defined as the frequency offset. Thus, the voltage of the triangular driving waveform is mapped to the frequency of the LFM-CW laser with a frequency tuning efficiency of $-f_cf_s/2V_\pi\Delta f$. The frequency tuning range and chirp rate of the linearly frequency-modulated laser can be calculated to be $|s_{pp}f_cf_s/2V_\pi\Delta f|$ and $|s_{pp}f_cf_s^2/V_\pi\Delta f||$, respectively, where s_{pp} is the peak-to-peak amplitude of the triangular driving waveform.

Supplementary note II: SBS suppression based on LFM-CW laser

Figure S2a illustrates the schematic diagram of the SBS-induced impairment on the transmitted wireless communication signal. Insets a-f depict the mechanism of the Brillouin scattering-induced signal-to-noise ratio (SNR) degradation. In the conventional long-haul high-power analog radio-over-fiber (A-RoF) link, the optical carrier is first intensity-modulated by a broadband wireless communication signal via using a Mach-Zehnder modulator (MZM), and then boosted by using an optical amplifier (as shown in Fig. S2b-c). The optically-carried signal is then injected into the single-mode fiber (SMF) and dispersion compensating fiber (DCF). When the injected optical power exceeds the Brillouin threshold, the forward-propagating optical carrier interacts with the acoustic-phonon for generating a backward-propagating Stokes wave within the phonon lifetime, as shown in Fig. S2a. The interference between the Stokes

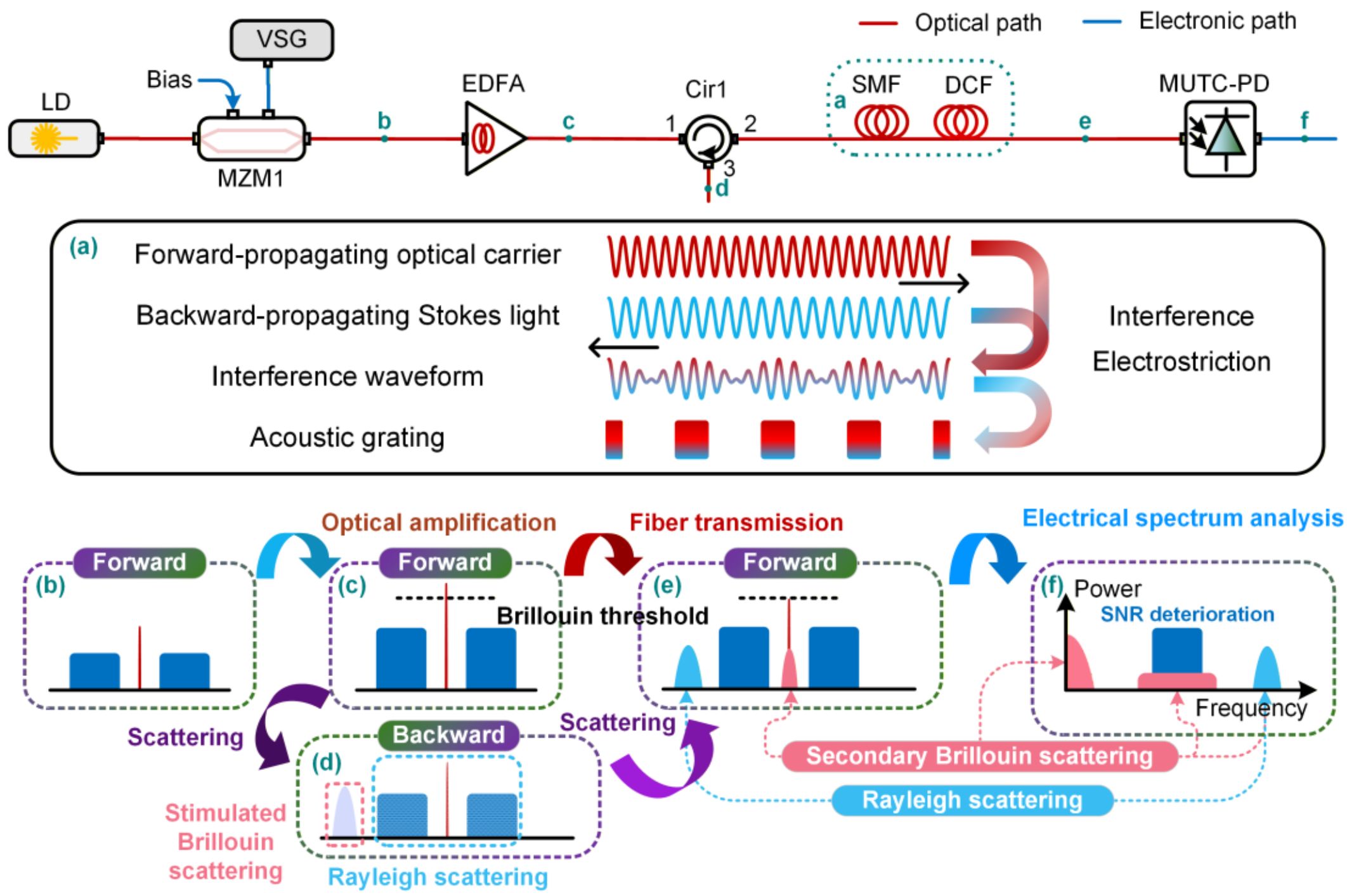

Fig. S2. Schematic diagram of the SBS-induced impairment on the transmitted wireless communication signal. a. A large portion of the forward-propagating optical carrier power is transferred to the forward anti-Stokes light via the SBS and the secondary Brillouin scattering processes, introducing a large noise to the recovered wireless communication signal and deteriorates the SNR of the transmitted signal (as shown in **b-f**).

wave and the optical carrier generates a stable acoustic grating via electrostriction effect. This grating reinforces the coupling between the optical carrier and the Stokes wave, which converts an ever-larger fraction of the forward-propagating optical carrier power into the backward-propagating Stokes light. Consequently, a strong Stokes light is generated in the backward direction, as shown in Fig. S2d. Then, the backward-propagating Stokes light generates anti-Stokes light in the forward direction, which is at the vicinity of the optical carrier. This process is referred to as secondary Brillouin scattering, as shown in Fig. S2e.

At the remote end, the anti-Stokes light due to the secondary Brillouin scattering is superimposed on the forward-propagating optical carrier, and then beats with the modulation sidebands, introducing a large noise to the wireless communication signal from the modified uni-traveling carrier photodiode (MUTC-PD). Except for the above noise, the power of the wireless communication signal derived from the beating between the optical carrier and the modulation sidebands is greatly reduced since most of the forward-propagating optical carrier power is transferred to the backward Stokes light and the forward anti-Stokes light via the SBS process and the secondary Brillouin scattering effect, respectively. Consequently, severe SNR deterioration occurs, restricting the performance of the high-power A-RoF link. Moreover, forward-propagating Stokes light is generated via Rayleigh scattering of the backward-propagating Stokes light as shown in Fig. S2e. As a result, the noise floor at the vicinity of the Brillouin frequency shift is elevated due to the beating between the forward Stokes light and the optical carrier, and that between the forward Stokes light and the anti-Stokes light, which largely degrades the signal transmission quality at the vicinity of this frequency, as shown in Fig. S2f. Besides, the noise floor near direct current (DC) is also raised due to beating between the optical carrier and the anti-Stokes light. Therefore, it is of great importance to suppress the SBS and the secondary Brillouin scattering in the long-haul high-power A-RoF link.

In the proposed linearly frequency-modulated laser-based long-haul high-power

A-RoF link, the SBS-induced SNR deterioration is mitigated via using a chirped optical carrier. Herein, the SBS threshold enhancement arises from two complementary physical mechanisms:

(1) Spectral broadening (frequency-domain effect). When the optical carrier is linearly frequency-modulated over a tuning bandwidth Δf_{tuning}, its spectrum is effectively broadened over that range. The Brillouin interaction, however, is intrinsically a narrowband process. The Brillouin gain spectrum in standard single-mode fiber (SSMF) at 1550 nm has a full-width at half-maximum (FWHM) bandwidth $\Delta\nu_B$ of approximately 10 – 30 MHz. The spectral overlap between the broadened pump spectrum and the narrow Brillouin gain spectrum is therefore reduced by a factor approximately equal to $\Delta\nu_B/\Delta f_{tuning}$. This spectral overlap reduction lowers the effective Brillouin gain coefficient, yielding a threshold enhancement that scales as $\Delta f_{tuning}/\Delta\nu_B$.

(2) Dynamic suppression (time-domain effect). Beyond the spectral argument, the temporal dynamics of the Brillouin interaction provide an additional suppression mechanism. The build-up of the acoustic grating in a long optical fiber is not instantaneous, but requires a finite time to reach steady-state amplitude. In long optical fibers, this build-up time has been experimentally characterized to range from sub-microseconds to milliseconds, depending on the pump power and feedback conditions. When the residence time is comparable to or shorter than the SBS build-up time, the acoustic grating cannot reach its steady-state amplitude before the pump frequency has swept out of the gain bandwidth, resulting in additional suppression that scales with the chirp rate γ.

Mathematically, the effective Brillouin gain under chirped excitation can be modeled as

$$g_B^{eff} = g_B \cdot \frac{\Delta\nu_B}{\Delta f_{tuning}} \cdot F\left(\gamma, \tau_{SBS}\right) \tag{7}$$

where g_B is the peak Brillouin gain coefficient for a CW carrier. $F(\gamma, \tau_{SBS}) \leq 1$ is the dynamical reduction factor, which can be expressed as the ratio of the residence time

τ_{res} to the SBS build-up time τ_{SBS}

$$F(\gamma) = \frac{\tau_{res}}{\tau_{SBS}} = \frac{2\Delta\nu_B}{\gamma\tau_{SBS}} \tag{8}$$

Substituting $F(\gamma, \tau_{SBS})$ into (7), the SBS threshold enhancement factor η is obtained as

$$\eta = \frac{P_{th}^{LFM}}{P_{th}^{CW}} = \frac{\Delta f_{tuning}\gamma\tau_{SBS}}{2(\Delta\nu_B)^2} \tag{9}$$

It can be seen from (9) that the threshold enhancement η increases with both the sweep bandwidth Δf_{tuning} and the chirp rate γ, confirming that broader sweep range and higher chirp rate effectively disrupt the acoustic grating build-up in both the frequency and time domains. It thereby provides stronger SBS suppression.

In the experiment, the frequency tuning range and the sweep period of the LFM laser are measured to be 150 MHz and 202.6 ns. The residence time can be further calculated to be 40.5 ns, which is comparable to the SBS build-up time reported for long-fiber systems under practical conditions. This implies that the acoustic grating cannot fully establish itself before the pump frequency moves out of the Brillouin gain bandwidth. Taking the SBS build-up time τ_{SBS} to be in the sub-microsecond range, the Brillouin threshold enhancement is calculated to be 12 dB. The residual discrepancy between the theoretical prediction and the experimental value can be attributed to several factors. First, the model assumes an ideal Lorentzian Brillouin gain spectrum and a well-defined SBS build-up time τ_{SBS}, whereas the actual SBS dynamics in a 11.8-km fiber link (comprising both SMF and DCF) involve distributed variations in Brillouin gain parameters along the fiber. Second, the DCF segment exhibits a different Brillouin frequency shift and gain coefficient compared to the SMF, which complicates the effective interaction. Third, the chirp linearity and phase noise of the synthesized LFM laser introduce minor deviations from the ideal chirp profile assumed in the model. Nevertheless, the quantitative model successfully captures the correct order of magnitude and the scaling behavior of the threshold enhancement with the sweep parameters, confirming that the observed 11-dB enhancement arises from the joint

action of spectral broadening in the frequency domain and dynamic suppression in the time domain—both enabled by the high chirp rate and broad sweep bandwidth of the time-phase synthesis laser.

Supplementary note III: Operation principle of fiber delay measurement and breakpoint localization

Both the fiber delay measurement and the fiber breakpoint localization share the same fundamental task, i.e., determining the realistic time-of-flight along the optical fiber. The key technique of the proposed optical fiber sensing scheme lies in optical de-chirping. Figure S3 presents the operation principle of optical de-chirping and unambiguous delay measurement range extension based on the linearly frequency-modulated laser. The heterodyne detection yields a continuous low-frequency signal with a stable frequency component of $\Delta f = 2\pi\gamma\Delta\tau$, where γ and $\Delta\tau$ are the chirp rate of the linearly frequency-modulated laser and the time delay between the local oscillator light and the reflected probe light. Herein, $\Delta\tau$ can be further obtained via frequency discrimination. Nevertheless, the period of the linearly frequency-modulated laser is restricted by the round-trip time of the ring-cavity laser to be smaller than 1 μs. Besides, the round-trip time delay in the A-RoF link is generally larger than 10 μs (corresponding

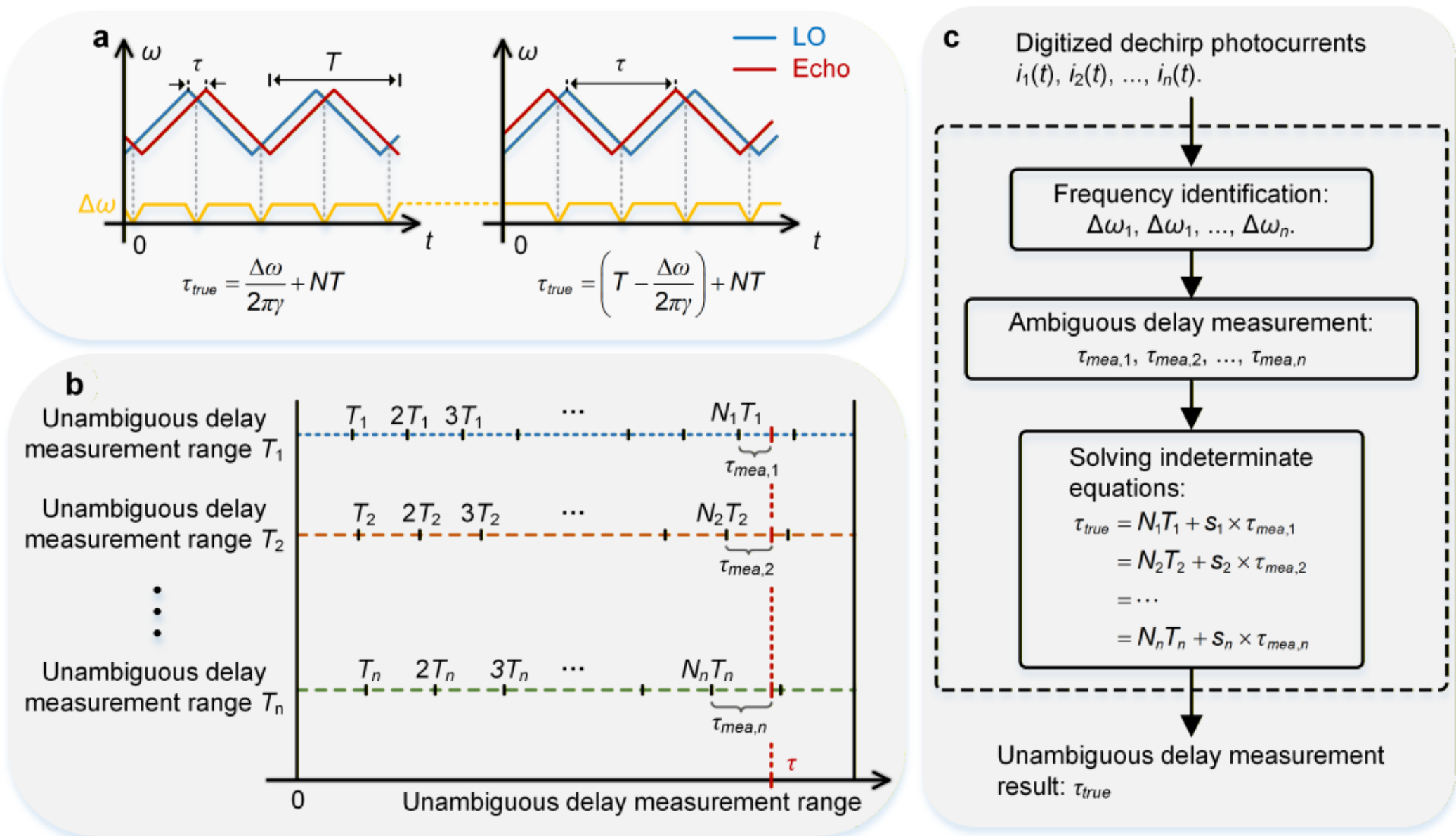

Fig. S3. Operation principle of optical de-chirping and Vernier effect-based unambiguous delay measurement range extension. a Heterodyne optical de-chirping. The reflected LFM-CW probe light beats with the LO light to produce a low-frequency signal, where the signal frequency is proportional to the round-trip delay. **b** Inter-period unambiguous delay measurement range extension scheme based on the Vernier effect. **c** Proposed ambiguity-resolution algorithm for resolving the intra-period ambiguity.

to a fiber length of ~1 km). If the linearly frequency-modulated laser is directly utilized for fiber delay measurement and fiber breakpoint localization, a serious aliasing will occur in the de-chirped signal. Moreover, owing to the intra-period symmetry of the triangular waveform, an ambiguity in the measured time-of-flight arises even within a single period, as shown in Fig. S3a. Consequently, the unambiguous delay measurement range is constrained using a single linearly frequency-modulated laser. To address this issue, we propose a Vernier effect-based unambiguous delay measurement range extension scheme, as shown in Fig. S3b. Through performing repeated time delay measurement via utilizing linearly frequency-modulated lasers with different chirp rates, the unambiguous delay measurement range is enlarged. The intra-period ambiguity is also resolved via using an ambiguity-resolution algorithm displayed in Fig. S3c. As a result, a high-precision fiber delay measurement can be obtained.

Mathematically, the angular frequency $\omega_{optical}(t)$ of the linearly frequency-modulated laser in a single period T is expressed as

$$\omega_{optical}(t)=\begin{cases}\omega_c+2\pi\gamma t, 0\le t\le \dfrac{T}{2}\\ \omega_c+B-2\pi\gamma\left(t-\dfrac{T}{2}\right), \dfrac{T}{2}<t\le T\end{cases} \tag{10}$$

where ω_c and $B=\pi\gamma T$ represent the initial angular frequency and the angular frequency tuning range of the linearly frequency-modulated laser, respectively. The optical phase of the linearly frequency-modulated laser is the integral of the optical frequency, which is expressed as

$$\begin{aligned}\phi(t)&=j\int_{-\infty}^{t}\omega_{optical}(\zeta)d\zeta\\&=\begin{cases}j\left(\omega_c t+\pi\gamma t^2+\varphi_0\right), 0\le t\le \dfrac{T}{2}\\ j\left(\left(\omega_c+2B\right)t-\pi\gamma t^2-\dfrac{1}{2}BT+\varphi_0\right), \dfrac{T}{2}\le t\le T\end{cases}\end{aligned} \tag{11}$$

where φ_0 represents the initial phase of the linearly frequency-modulated laser, and is expressed as

$$\varphi_0=\int_{-\infty}^{0}\omega_{optical}(t)dt \tag{12}$$

The optical field of the linearly frequency-modulated laser is expressed as $E(t) = A\exp(j\varphi(t))$, where A denotes the amplitude of the optical field. In the proposed fiber delay measurement scheme, the optical field of the LO light is expressed as $E_{ref}(t) = E(t)$. The reflected probe light with a time delay τ is expressed as

$$E_{mea}(t) = \begin{cases} \alpha A\exp\left(j\left(\omega_c(t-\tau)+\pi\gamma(t-\tau)^2+\varphi_0\right)\right), 0 \le t \le \dfrac{T}{2} \\ \alpha A\exp\left(j\left(\left(\omega_c+2B\right)(t-\tau)-\pi\gamma(t-\tau)^2-\dfrac{1}{2}BT+\varphi_0\right)\right), \dfrac{T}{2} \le t \le T \end{cases} \tag{13}$$

where α denotes the amplitude attenuation coefficient of the fiber link. Through heterodyne detection of the time-delayed echo light and the local oscillator light, the output photocurrent from PD2 is expressed as

$$\begin{aligned} i(t) &= real\left\{\eta \cdot E_{mea}(t) \cdot E_{ref}^{*}(t)\right\} \\ &= \begin{cases} \eta\alpha^2 A^2 \cos\left(2\pi\gamma\tau t-\pi\gamma\tau^2+\omega_c\tau\right), 0 \le t \le \dfrac{T}{2} \\ \eta\alpha^2 A^2 \cos\left(2\pi\gamma\tau t-\pi\gamma\tau^2-(\omega_c+2B)\tau\right), \dfrac{T}{2} \le t \le T \end{cases} \end{aligned} \tag{14}$$

The instantaneous frequency $\Delta\omega = 2\pi\gamma\tau$ of the de-chirped signal can be deduced via utilizing the Hilbert-transform-based phase extraction method or the Fourier-transform-based phase spectrum analysis technique. Due to that the realistic delay time τ_{ture} is larger than the period T of the probe light, an unavoidable intra-period and inter-period ambiguity appears. Herein, the measured time delay $\tau_{mea} = \tau$ is expressed as

$$\tau_{mea} = \begin{cases} \tau_{true} - NT, NT < \tau_{true} < NT + \dfrac{T}{2} \\ (N+1)T - \tau_{true}, NT + \dfrac{T}{2} < \tau_{true} < (N+1)T \end{cases} \tag{15}$$

where N is a positive integer. It can be observed that the measured time delay τ_{mea} is limited to the range of $[0, T/2)$ as an aliased value.

To perform an unambiguous measurement of such a large time delay, a Vernier effect-based unambiguous delay measurement range extension scheme is proposed and demonstrated. Benifiting from the reconfigurability of the proposed LFM-CW laser (as detailed in Supplementary Note I), the periodic ambiguity time delay measurement

described in (15) can be conducted multiple times by adjusting the LFM-CW period. A series of linearly frequency-modulated lasers with different periods of T_i (i = 1, 2, ..., n) are generated by adjusting the driving signal. Conrespondingly, a group of time delays $\tau_{mea,i}$ (i = 1, 2, ..., n) are mesured. According to (15), each $\tau_{mea,i}$ can be used to construct a equation for resolving the unambiguous value τ_{ture}, i.e.,

$$\tau_{true} = nT_i + s \times \tau_{mea,i} \tag{16}$$

where $s \in \{+1,-1\}$ is a binary choice variable. Then, the realistc time delay can be obtained by solving the time-delay measurement equation group, i.e.,

$$\begin{aligned} \tau_{true} &= N_1 T_1 + s_1 \times \tau_{mea,1} \\ &= N_2 T_2 + s_2 \times \tau_{mea,2} \\ &\;\vdots \\ &= N_n T_n + s_n \times \tau_{mea,n} \end{aligned} \tag{17}$$

which is the core of the proposed ambiguity-resolution algorithm depicted in Fig. S3c. In (17), the $\tau_{mea,i}$ and T_i are known, while N_i and s_i are the variables to be determined. Because N_i is a positive integer and s_i is a binary choice variable, the τ_{ture} in (17) possesses a unique solution within the unambiguous measurement range of the system. The detailed solution to the equation set is given below.

First, the first equation is extracted from the system using the pairwise intersection method.

$$N_l T_l + s_l \times \tau_{mea,l} = N_m T_m + s_m \times \tau_{mea,m} \tag{18}$$

Initially, all combinations of the binary choice variables $s \in \{+1,-1\}$ are considered. For each specific assignment of s, these become known coefficients in the equation. The remaining unknowns N_l and N_m are then resolved through exhaustive enumeration, retaining only those pairs that satisfy the error constraints

$$\left| \left(N_l T_l + s_l \times \tau_{mea,l} \right) - \left(N_m T_m + s_m \times \tau_{mea,m} \right) \right| < \delta\tau \tag{19}$$

where $\delta\tau$ denotes the delay measurement error of the system. Once N_l and N_m are resolved, the true time delay τ_{ture} can be calculated by (17). It should be noted that the pairwise intersection method yields four possible solutions for τ_{ture}, corresponding to

the four combinations of the binary choice variable $s \in \{+1,-1\}$. Among these, only one corresponds to the true value, while the remaining three are erroneous.

Subsequently, the second equation of the pairwise intersection method is considered. By repeating the above procedure, the second equation also yields four candidate values for τ_{ture}, again consisting of one true value and three spurious ones. It should be noted that the erroneous solutions arising from different equations are generally distinct, whereas the true value remains consistent across all equations. Therefore, the true time delay τ_{ture} can be identified by taking the common solution shared by both sets of equations.

In conclusion, it is evident that extracting the true unambiguous time delay from the set of potentially ambiguous candidates requires repeated measurements using at least three distinct frequency-modulation periods of the LFM-CW laser. For most practical scenarios, three periods are sufficient to determine the correct unambiguous delay, as demonstrated in Section 2.3(B) and Supplementary Note VI. In the exceptional case where three periods prove inadequate, the issue can be readily resolved by incorporating additional modulation periods and repeating the aforementioned solving procedure.

It should be noted that, when two linearly frequency-modulated lasers with periods of T_p and T_q are employed for time-delay measurement, the unambiguous delay measurement range of the proposed scheme is expressed as $T_pT_q/|T_q-T_p|$ based on the Vernier effect. Through properly setting the period T_i, the unambiguous delay measurement range can be adjusted to exceed the round-trip fiber delay. As a result, inter-period ambiguity is resolved. Furthermore, the intra-period ambiguity shown in (15) and Fig. S3(a) is eliminated using a dedicated scheme, which relies on the use of at least three LFM lasers and is illustrated in Fig. S3b and S3c. Thus, both the inter-period and intra-period ambiguities are completely resolved, and the unambiguous time-delay measurement range of the system is successfully extended.

Supplementary note IV: Operation principle of wireless sensing

Figure S4 presents the operation principle of the proposed wireless sensing scheme for target ranging. The wireless probe signal is generated using an optical heterodyne method. The wireless sensing subsystem is built upon the canonical radar-ranging architecture. At the receiving end, the echo signal from the target is mixed with a local oscillator signal for de-chirping using a microwave mixer. The output IF signal from the mixer carries both the range and velocity information of the target, which can be retrieved by discriminating the frequency components of the de-chirped waveform, as shown in Fig. S4b and c.

Mathematically, the LFM microwave signal generated at the remote base station is expressed as

$$s_{LFM}(t)=\begin{cases} A_{LFM}\cos\left(2\pi f_0 t+\pi k t^2\right), 0\le t\le \dfrac{T}{2} \\ A_{LFM}\cos\left(2\pi(f_0+2BW)t-\pi k t^2-BW\cdot\dfrac{T}{2}\right), \dfrac{T}{2}\le t\le T \end{cases} \tag{20}$$

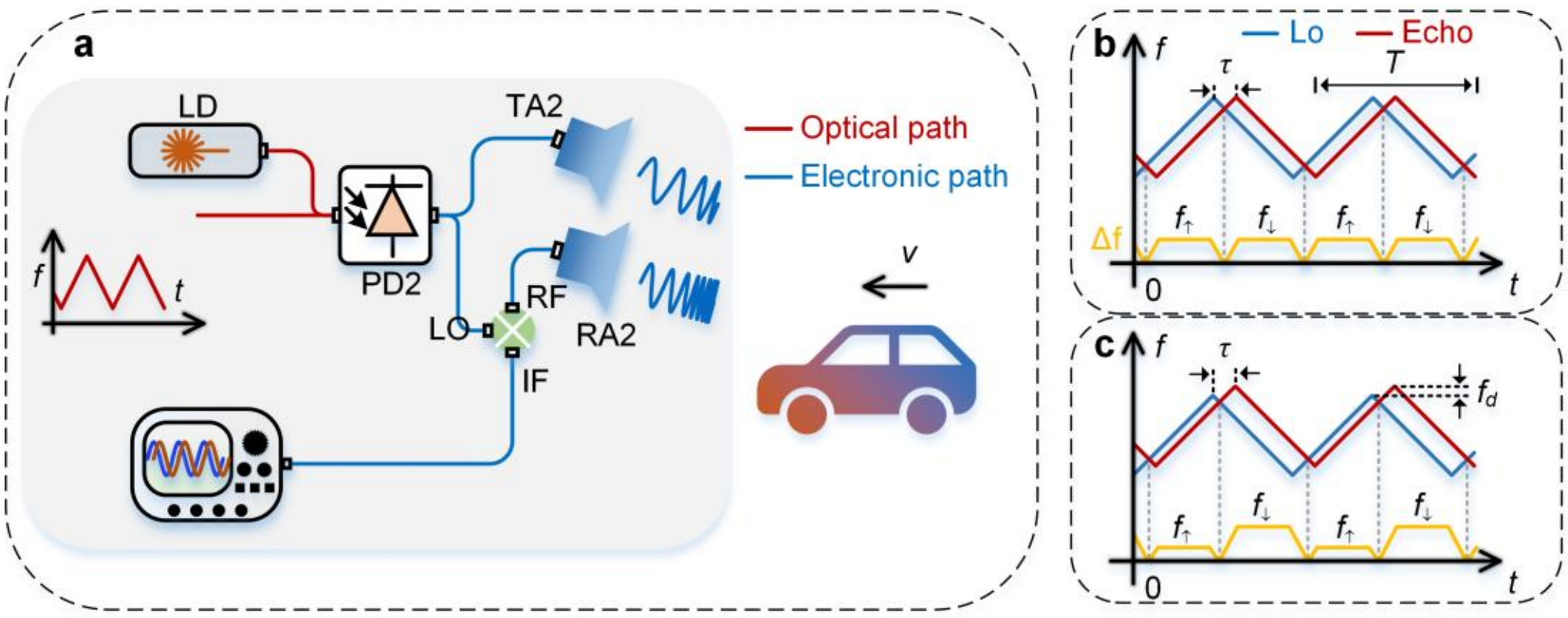

Fig. S4. Schematic diagram of wireless sensing. a Architecture of the radar-like wireless sensing scheme. The LFM-CW optical carrier is heterodyned with a frequency-offset LO light to generate an LFM microwave waveform for wireless sensing. For a stationary target, the echo signal is mixed with a copy of the original LFM microwave waveform. The output de-chirped signal possesses a constant-frequency component that is proportional to the round-trip time (as shown in **b**). When the target is in motion, Doppler shift imposes an additional frequency offset on the beat note. Distance and velocity of the target can be obtained by simultaneously measuring the up and down de-chirped signal, as depicted in **c**.

where A_{LFM} denotes the amplitude of the microwave signal. f_0, k, and T represent the initial frequency, chirp rate, and period of the linearly frequency-modulated microwave signal, respectively. $BW = kT/2$ is the frequency tuning range of the linearly frequency-modulated signal. The linearly frequency-modulated microwave signal is then split via an electrical power divider into two portions, i.e., a local oscillator signal and a probe signal. For a stationary target, the reflected probe signal experiences a round-trip time delay of $\tau = 2nL/c$. L is the distance between the target and the wireless radar antenna. c and n are the light speed in vacuum and the refractive index of the propagation medium, respectively. When the target is in motion, an additional Doppler frequency shift of $f_D = 2vnf_0/c$ is introduced into the echo signal, where v is the radial velocity of the target. Thus, the echo signal is expressed as

$$s_{echo}(t) = \begin{cases} A_{LFM}\cos\left(2\pi f_0(t-\tau) + \pi k(t-\tau)^2 + 2\pi f_D t\right), 0 \le t \le \dfrac{T}{2} \\ A_{LFM}\cos\left((2\pi(f_0 + 2BW)(t-\tau) - \pi k(t-\tau)^2 + 2\pi f_D t\right), \dfrac{T}{2} \le t \le T \end{cases} \quad (21)$$

When the local oscillator signal and the echo signal are injected into the local oscillator and the RF ports of the mixer, frequency-domain pulse compression is realized. The output IF signal from the mixer is expressed as

$$s_{dechirp}(t) \propto \begin{cases} \cos\left(2\pi(k\tau - f_D)t + 2\pi f_0\tau - \pi k\tau^2\right), 0 \le t \le \dfrac{T}{2} \\ \cos\left(2\pi(k\tau + f_D)t - 2\pi(f_0 + \pi kT)\tau + \pi k\tau^2\right), \dfrac{T}{2} \le t \le T \end{cases} \quad (22)$$

It can be observed that the de-chirped signal obtained from the up-chirp and down-chirp segments of the linearly frequency-modulated waveform exhibit a frequency difference due to the Doppler effect. Through performing precise frequency discrimination on these two signals, both the distance and velocity of the target can be determined as

$$L = \frac{c}{4nk}\cdot\left(f_{up} + f_{down}\right)$$
$$v = \frac{c}{4nf_0}\cdot\left(f_{down} - f_{up}\right) \quad (23)$$

where f_{up} and f_{down} represent the de-chirped frequency from the up- and down-chirp segments, respectively. Furthermore, a synthetic aperture radar (SAR) imaging can be achieved via integrating range–Doppler data across multiple spatial positions. In this way, the wireless perception of objects in the environment can be further enhanced by leveraging the mature radar imaging algorithm.

Supplementary note V: Reconfigurability of LFM-CW laser

The frequency tuning range and period of the LFM-CW laser can be flexibly reconfigured by adjusting the repetition frequency and peak-to-peak voltage of the drivig signal. The detailed principle of the voltage-to-frequency mapping is provided in Supplementary note I. Fig. S5 illustrates LFM-CW lasers with various frequency tuning ranges and repetition frequencies. Table S1 summarizes the setting parameters of the triangular driving waveforms and the obtained frequency tuning ranges of the LFM-CW lasers. It can be seen from Fig. S5 and Table S1 that the proposed scheme can generate a highly reconfigurable LFM-CW laser with a wide tuning range in both frequency and temporal period. Moreover, the frequency tuning range of the LFM-CW laser rigorously obeys the mapping relation derived in Supplement note I. The minor deviations are attributed to the RF loss between the AWG and the PM.

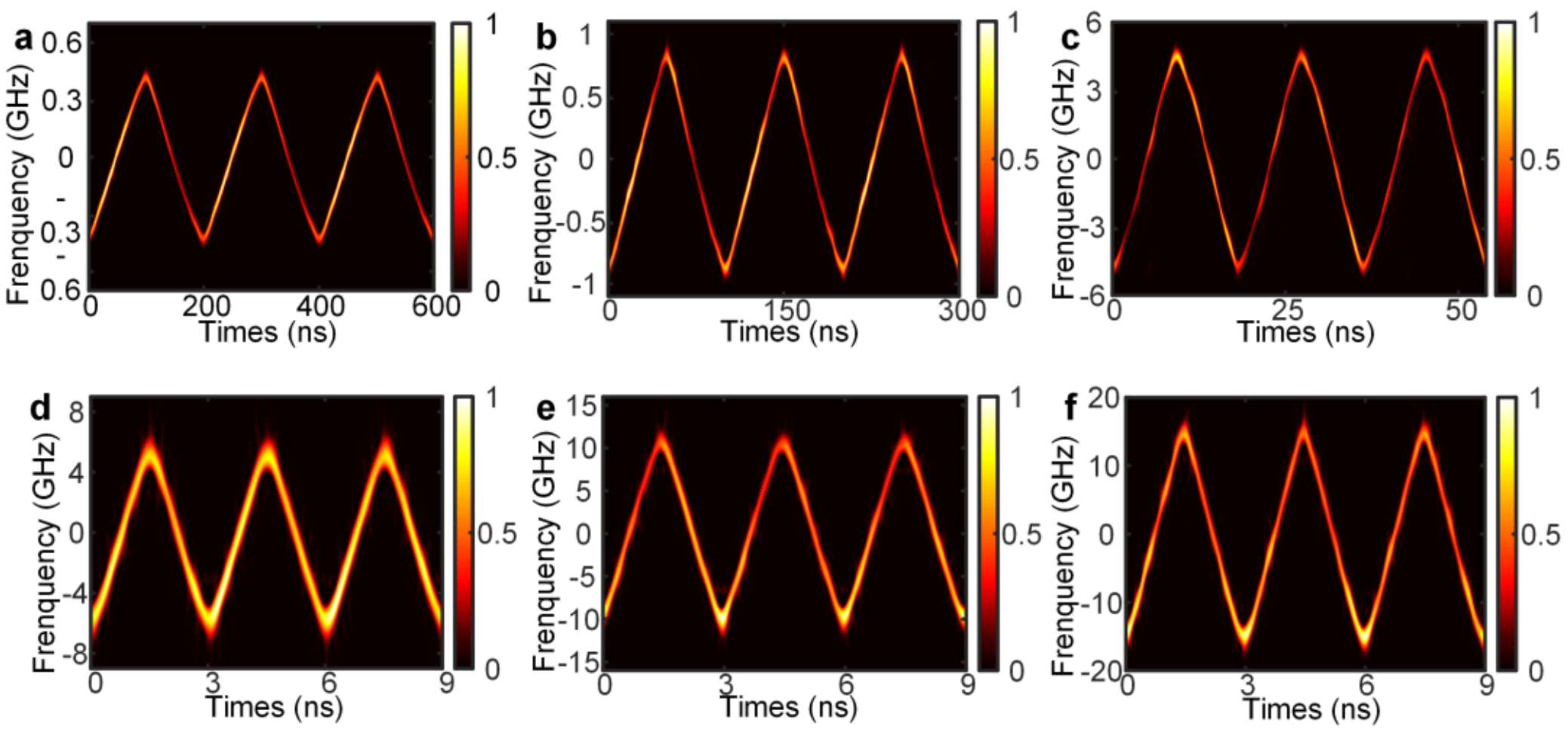

Fig. S5. Time-frequency distributions of LFM-CW lasers. The frequency tuning range and repetition frequency of the LFM-CW laser are flexibly adjusted by varying the peak-to-peak voltage and period of the triangular driving waveform.

Table S1. Detailed setting parameters and experiment results

Subplots in Fig. S5	Driving signal		Frequency tuning range	
	Peak-to-peak voltage	Repetition frequency	Therotical	Realistic
**a**	5.0 V	4.974 MHz	0.854 GHz	0.850 GHz
**b**	5.0 V	9.976 MHz	1.856 GHz	1.850 GHz
**c**	5.0 V	54.976 MHz	10.23 GHz	10.00 GHz
**d**	2.4 V	334.941 MHz	12.16 GHz	12.00 GHz
**e**	4.4 V	334.941 MHz	22.30 GHz	22.00 GHz
**f**	6.2 V	334.941 MHz	31.43 GHz	31.00 GHz

Supplementary note VI: Theoretical analysis of the coherence-sweep bandwidth trade-off in the time-phase synthesis laser

1. Physical origins of coherence degradation

The coherence of a frequency-swept optical signal is fundamentally determined by two factors, i.e., the intrinsic phase noise of the laser source and the fidelity of the frequency sweep. For an ideal LFM carrier with no phase noise, the optical field can be expressed as

$$E(t) = E_0 \exp\left(j2\pi \left(\nu_0 t + \frac{1}{2}\gamma t^2 \right) \right) \tag{24}$$

where ν_0 is the starting frequency and γ is the chirp rate. In this ideal case, the optical signal is perfectly coherent because the phase evolves deterministically.

In practice, however, the phase of the LFM signal contains both deterministic and stochastic components

$$\phi(t) = 2\pi \left(\nu_0 t + \frac{1}{2}\gamma t^2 \right) + \phi_{PN}(t) + \phi_{err}(t) \tag{25}$$

where $\phi_{PN}(t)$ is the stochastic phase noise inherited from the laser and the driving electronics. $\phi_{err}(t)$ is the deterministic phase error arising from nonlinearity in the frequency sweep.

The coherence of the optical signal is determined by how rapidly $\phi_{PN}(t)$ and $\phi_{err}(t)$ accumulate over time. The coherence time τ_{coh} is defined as the time over which the phase remains predictable within a certain tolerance. For a swept signal, this translates to a coherence length $L_c = c \cdot \tau_{coh}$, which represents the maximum round-trip distance over which the signal can maintain phase coherence for interferometric applications such as fiber sensing.

When the frequency sweep range Δf_{tuning} is increased—for example, by increasing the modulation index s_{pp} or by reducing the frequency offset Δf—the coherence degrades through three distinct physical mechanisms.

(1) Phase noise amplification. The frequency tuning efficiency of the time-phase synthesis laser is characterized by a mapping coefficient $K=f_c f_s/(2V_\pi \Delta f)$, which converts the driving voltage to the laser frequency excursion. When the sweep range is increased by raising the modulation index, the same coefficient K amplifies any phase noise present on the driving signal. Since the instantaneous linewidth δv_{inst} scales with the power spectral density of the frequency noise, it can be deduced as

$$\delta v_{inst} = \delta v_0 + K^2 \cdot \delta v_{AWG} \tag{26}$$

where δv_0 is the intrinsic linewidth of the cavity (determined by the cavity Q-factor and the EDFA noise). δv_{AWG} characterizes the phase noise of the AWG. The K^2 dependence arises because the frequency noise power is proportional to the square of the voltage-to-frequency conversion gain. A wider sweep (larger s_{pp} or smaller Δf) increases K, thereby amplifying the AWG phase noise and broadening the instantaneous linewidth, which directly reduces the coherence time.

(2) Nonlinear chirp and phase error accumulation. When the driving voltage swing approaches or exceeds the linear regime of the phase modulator, the induced phase shift deviates from the ideal linear electro-optic response. This deviation introduces a time-dependent frequency error $\varepsilon(t)$ into the chirp profile

$$v(t) = v_0 + \gamma t + \varepsilon(t) \tag{27}$$

The error $\varepsilon(t)$ is typically negligible for small modulation indices but grows superlinearly with the modulation index once the modulator enters its nonlinear regime. The accumulated phase error over the sweep period T is

$$\Delta\phi_{err} = 2\pi \int_0^T \varepsilon(t)\,dt \tag{28}$$

This phase error acts as an additional source of phase uncertainty that limits the coherence length. The effective linewidth contribution from the nonlinear chirp is approximately $\delta v_{nonlin} \approx \Delta\phi_{err}/T$, leading to a coherence length

$$L_c \approx \frac{c}{\pi\left(\delta v_{inst} + \delta v_{nonlin}\right)} \tag{29}$$

Since $\varepsilon(t)$ grows superlinearly with the modulation index (and thus with Δf_{tuning}), the coherence length degrades faster than linearly as the sweep range is increased.

(3) Mode-hopping instability. Reducing the frequency offset Δf offers a dramatic way to enhance the frequency sweep range of the laser. Nevertheless, when the driving repetition frequency Δf approaches an integer multiple of the cavity FSR (i.e., $\Delta f = f_s - kf_c \rightarrow 0$), the laser operates in a regime where the timing of the circulating optical field is nearly synchronized with the periodic driving waveform. In this regime, the system becomes highly susceptible to environmental perturbations. Even minute fluctuations in temperature or mechanical vibration cause the cavity length to drift, which shifts f_c and thus alters Δf. This can induce mode-hopping, which introduces discontinuous phase jumps that completely destroy the phase continuity required for long-coherence applications. This mechanism imposes a practical upper bound on the achievable sweep range for coherence-sensitive applications.

2. Quantitative relationship between coherence and sweep parameters

Combining the above mechanisms, the coherence length of the time-phase synthesis laser can be expressed as a function of the sweep bandwidth Δf_{tuning}

$$L_c\left(\Delta f_{tuning}\right) \approx \frac{c}{\pi\left(\delta v_0 + \alpha\left(\Delta f_{tuning}\right)^2 + \beta\left(\Delta f_{tuning}\right)^4\right)} \tag{30}$$

The three terms in the denominator correspond to the three mechanisms described above. δv_0 represents the fundamental linewidth of the cavity, independent of the sweep parameters. $\alpha(\Delta f_{tuning})^2$ represents the phase noise amplification contribution. $\beta(\Delta f_{tuning})^4$ represents the nonlinear chirp contribution.

Here, α and β are empirical coefficients that depend on the specific characteristics of the phase modulator and the AWG. These coefficients can be determined through independent calibration measurements of the chirp linearity and phase noise as a function of the modulation index. It can be seen from (30) that the coherence length decreases monotonically as the sweep bandwidth increases. The degradation becomes

more severe at larger bandwidths, as the modulation nonlinearity becomes increasingly pronounced.

Supplementary note VII: Fiber breakpoint localization

Figure S6 presents the experiment results for fiber breakpoint localization. Fig. S6a, d, and g show the time-frequency distribution of the employed linearly frequency-modulated probe light. It can be seen that the periods of the linearly frequency-modulated probe lights are 202.881 ns, 202.593 ns, and 202.265 ns, respectively, which are identical to the probe light employed in the fiber delay measurement. Figure S6b, e, and h show the time-frequency distribution of the de-chirped RF signal. It can be seen that the frequencies of the de-chirped signals are 46.260 MHz, 26.658 MHz, and 90.072 MHz, respectively. Then, the preliminary time-delay within half periods are calculated to be 39.57 ns, 20.82 ns, and 60.93 ns, respectively, as depicted in Fig. S6 c, f, and i. Through applying the Vernier effect-based unambiguous delay measurement range extension method and the intra-period ambiguity-resolution algorithm, an unambiguous round-trip delay of 100.2626 μs (equivalent to a fiber length of 10.02626 km) is achieved. The experiment result is very close to the total length of the SMF (10 km) employed in the proposed scheme. The tiny difference is attributed to the fiber delay in the EDFA and other passive fiber component such as the OC and the Faraday rotator mirror.

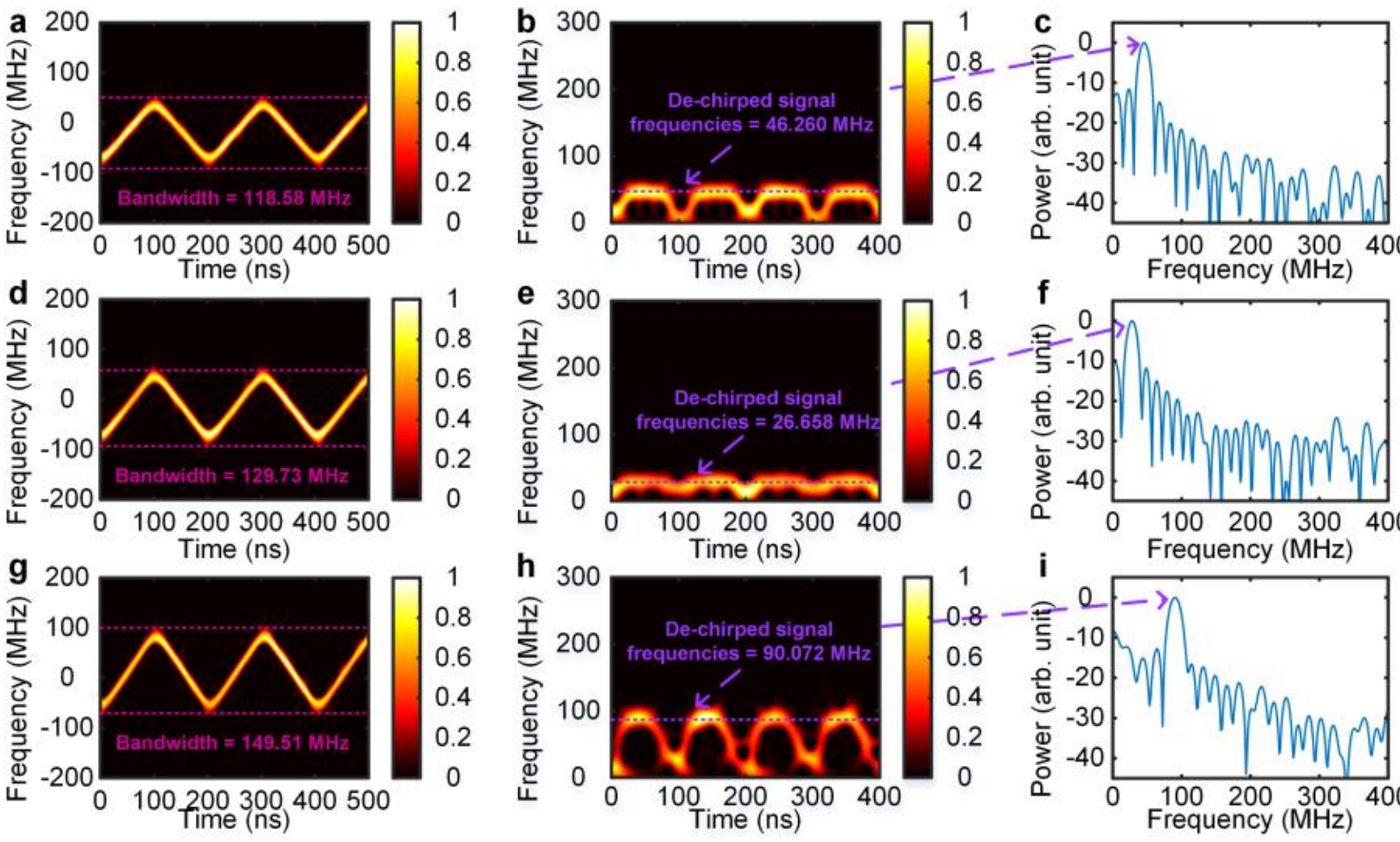

Fig. S6. Experimental results for fiber breakpoint localization. **a**, **d**, **g** Time-frequency distributions of the employed LFM-CW lasers. **b**, **e**, **h** Time-frequency distributions of the de-chirped signals. The frequency components of the de-chirped signals are exhibited in **c**, **f**, **i**.

Supplementary note VIII: Receiver sensitivity analysis of the wireless link

In the experiment, the real-time oscilloscope (Keysight UXR0594A) used for signal acquisition and demodulation operates at the maximum sampling rate and the minimum vertical resolution with front-end attenuation active. Under these operating conditions, the effective noise floor of the measurement system is determined by the combined effect of the front-end attenuation, the analog-to-digital converter (ADC) quantization noise, and the thermal noise of the input stage. Based on system calibration, the equivalent input-referred noise spectral density is measured to be approximately -125 dBm/Hz. Thus, the integrated noise power over the 135 MHz signal bandwidth is

$$P_{noise} = -125 + 10\lg\left(135 \times 10^{6}\right) = -43.7\text{dBm} \quad (31)$$

For a 64-QAM modulated wireless communication signal, the required signal-to-noise ratio is approximately 25 dB. Therefore, the minimum detectable signal power is calculated to be

$$P_{signal} = P_{noise} + \text{SNR} = -18.7\text{dBm} \quad (32)$$

This theoretical estimate of approximately -18.7 dBm is in excellent agreement with our experimentally observed sensitivity limit.

To provide direct experimental evidence for the receiver sensitivity, we performed systematic EVM measurements as a function of the received RF power. The experiment results are summarized in Table R2

Table R2 Measured EVM versus the received RF power.

Received Power (dBm)	EVM (%)	Status
-13	3.4%	Within 3GPP 8% limit
-15	4.2%	Within 3GPP 8% limit
-17	5.4%	Within 3GPP 8% limit
-19	8.3%	Exceeds 3GPP 8% limit
-21	9.1%	Exceeds 3GPP 8% limit

The measured EVM at -17 dBm is 5.4%, which is well below the 3GPP 8%

threshold for 64-QAM wireless communication signal. As the received power increases, the EVM decreases monotonically, consistent with the expected improvement in SNR. The 3GPP 8% threshold is reached at approximately -19 dBm, which agrees well with the theoretically calculated sensitivity limit of -18.7 dBm. The sensitivity limit could be further improved by optimizing the experimental setup, such as reducing the front-end attenuation of the oscilloscope or employing a lower-noise signal analyzer.
